# Subjective Identity Concealability and the Consequences of Fearing Identity-Based Judgment

**DOI:** 10.1177/01461672211010038

**Published:** 2021-04-23

**Authors:** Joel M. Le Forestier, Elizabeth Page-Gould, Calvin K. Lai, Alison L. Chasteen

**Affiliations:** 1 University of Toronto, Ontario, Canada; 2 Washington University in St. Louis, MO, USA

**Keywords:** concealable identities, stigma, intergroup anxiety, social identity threat, lay beliefs

## Abstract

In intergroup contexts, people may fear being judged negatively because of an identity they hold. For some, the prospect of concealment offers an opportunity to attenuate this fear. Therefore, believing an identity is concealable may minimize people’s fears of identity-based judgment. Here, we explore the construct of subjective identity concealability: the belief that an identity one holds is concealable from others. Across four pre-registered studies and a set of internal meta-analyses, we develop and validate a scale to measure individual differences in subjective identity concealability and provide evidence that it is associated with lower levels of the psychological costs of fearing judgment in intergroup contexts. Open materials, data, and code for all studies, pre-registrations for Studies 1–4, and online supplementary materials can be found at the following link: https://osf.io/pzcf9/.

Social scientists have long been concerned with the concealment of stigmatized identities. Indeed, early conceptualizations of stigma placed concealment among its most fundamental features (Becker, 1963; Goffman, 1963) and a large empirical literature on concealment has since emerged. Most of this literature has focused on identities that are assumed to be concealable by researchers such as homosexuality (e.g., S. W. Cole et al., 1996; Goh et al., 2019; Jackson & Mohr, 2016; Quinn et al., 2017; Riggle et al., 2017; Ullrich et al., 2003) or having had an abortion (e.g., Major & Gramzow, 1999). Relatively less is known about how concealable people believe their own identities are or about the consequences of these beliefs. In the present paper, we therefore develop and validate a measure of how concealable people believe their own identities are and test a hypothesis concerning one potential consequence of differences in such a belief: fear of judgment in intergroup contexts.

## Subjective Identity Concealability

We refer to the extent to which someone believes they are able to conceal an identity as subjective identity concealability. Subjective identity concealability concerns a person’s expected outcome of concealment, which is likely informed by factors including how concealable they believe an identity is in general, how skillful at concealing they believe themself to be, and more. This differs from previous approaches to studying concealment in that it does not label entire categories of identities as concealable or not, but rather considers individuals’ beliefs about the concealability of their own identities.

We argue that people who believe an identity they hold is concealable should be less susceptible to the costs of fearing intergroup judgment. We argue this is because a belief that an identity one holds is concealable may guide people to interpret their interactions in a way that is consistent with this belief.

## Lay Beliefs

Support for this hypothesis is drawn from research on lay beliefs. Beliefs shape people’s experiences by providing them with frameworks with which to make sense of the world around them (Levy et al., 2006). People then interpret their experiences in a way that is consistent with the framework provided by their belief (Levy et al., 2001). The belief that one’s identity is concealable may thus frame a person’s experiences in intergroup contexts. Because perception is a precondition for judgment, a belief in one’s own ability to conceal an identity may equip that person with a sense of imperviousness from judgment based on that identity. Therefore, such a belief may render people less vulnerable to the threat of being judged negatively based on that identity.

## Psychological Consequences of Fearing Intergroup Judgment

Fearing identity-based judgment can evoke social identity threat, which arises when one worries about being judged on the basis of a group identity (Steele et al., 2002), and intergroup anxiety, which arises when someone anticipates consequences relating to an intergroup interaction (Stephan & Stephan, 1985). Among members of stigmatized groups, these effects have been linked to negative outcomes including decreased performance in school and at work (Spencer et al., 2016), avoidance of stereotype-relevant domains (Steele & Aronson, 1995), reduced executive function (Richeson & Trawalter, 2005), negative emotions including fear, anger, and stress (Butz & Plant, 2006; Trawalter et al., 2012; Van Zomeren et al., 2007), less engagement in intergroup contact (E. R. Cole & Yip, 2008), and less effective cross-cultural communication (Ulrey & Amason, 2001).

## Identity Concealment

To date, psychologists have explored facets of concealment including general concealability (Pachankis et al., 2018), active concealment (Quinn et al., 2017), non-disclosure (Jackson & Mohr, 2016), outness (Mohr & Fassinger, 2000), and motivation (Mohr & Daly, 2008). In much of this work, greater levels of concealment (i.e., greater motivation and engagement in concealment and less disclosure and outness) have been linked to negative consequences in domains including mental and physical health (S. W. Cole et al., 1996; Pachankis et al., 2020; Quinn et al., 2017; Weisz et al., 2016), psychological well-being (Beals et al., 2009; Riggle et al., 2017), authenticity (Newheiser & Barreto, 2014; Riggle et al., 2017), and belonging (Newheiser & Barreto, 2014).

In general, these negative effects are more pronounced for those who actively engage in concealment behaviors, rather than people whose identities are simply unknown to those around them (Quinn et al., 2017). Consistent with this, these effects have been attributed to social isolation (Quinn et al., 2017), low levels of social support (Beals et al., 2009; Chaudoir & Fisher, 2010), and emotional suppression (Beals et al., 2009; S. R. Cole et al., 1996), all of which are more likely to result from engagement in concealment than from more passive forms of non-disclosure.

Some benefits of concealment have also been found. People who conceal stigmatized identities are evaluated more positively than those who do not (Sanchez & Bonam, 2009), receive more job interviews (Gaddis, 2015; Kang et al., 2016), and experience less discrimination (Pachankis & Bränström, 2018; c.f. Goh et al., 2019). These benefits have not escaped the notice of people with stigmatized identities. People who attempt to conceal their identities do so with the expectation that it will help them avoid discrimination (Kang et al., 2016).

## Present Work

In the present work, we argue that subjective identity concealability may shield people from the threatening experience of anticipating intergroup judgment. Drawing on insights from the concealment and lay beliefs literatures, we argue that the mere belief than an identity is concealable from others may attenuate the costs of fearing intergroup judgment. Because these effects emerge when the mere fear of intergroup judgment arises (Mallett et al., 2008), we argue that a mind-set of concealability may be sufficient to attenuate fears of identity-based judgment. This hypothesis compliments the current literature by focusing on beliefs rather than behaviors, individual differences within groups rather than group membership, and potential positive outcomes of a concealment-related process.

Across four pre-registered studies, we equip ourselves—and other researchers—to begin answering this and related questions by developing a scale to measure beliefs about concealability. Subsequently, in a set of internal meta-analyses, we test the proposition that people higher on subjective identity concealability should be less concerned with facing intergroup judgment. Study 1 used open-ended questions to understand reasons that participants believe various identities are easy or hard to conceal. These reasons were then used to develop a list of potential scale items. Studies 2 and 3 reduced the list of items to a final measure of subjective identity concealability. Study 4 assessed the scale’s convergent and discriminant validity, and Study 5 used an internal meta-analytic approach to assess the hypothesis that subjective identity concealability is associated with fewer costs of fearing identity-based judgment. These studies represent a pre-registered and multi-method approach to scale development, construct exploration, and hypothesis-testing.

## Study 1: Item Generation

In Study 1, we collected open-ended data about factors people believe influence the ease or difficulty of concealing an identity to inform the generation of scale items. We took a bottom-up approach to item generation with the goal of writing items that reflect people’s experiences.

### Method

#### Participants

Data were collected from 214 volunteers recruited through Project Implicit (https://implicit.harvard.edu; see Table 1 for demographics). All available data were used for each question, so no participants were excluded. Some participants did not provide responses to all questions. The number of observations included in each analysis is reported in the results.

**Table 1.** Demographic Details.

Source	Study 1	Study 2	Study 3	Study 4	Supp.^1^
	Project Implicit	Mechanical Turk	Project Implicit	Department pool	Project Implicit
*N*	214	298	1012	227	280
Age					
Mean (years)	37.42	35.19	32.93	19.11	39.74
*SD* (years)	13.28	9.30	14.96	1.93	15.51
No response (*n*)	19	2	6	0	2
Sex					
Female	69.00%	37.92%	68.32%	73.13%	52.86%
Male	29.00%	62.08%	31.58%	26.87%	46.07%
Other	2.00%	0.00%	0.10%	0.00%	1.07%
No response (*n*)	14	0	2	0	0
Ethnic Origin					
Aboriginal	0.00%	0.67%	0.20%	0.00%	0.36%
African	5.03%	5.72%	6.36%	3.08%	6.09%
Caribbean	0.50%	0.34%	1.59%	1.76%	1.43%
East/Southeast Asian	10.55%	4.04%	5.46%	49.34%	5.02%
European	54.27%	71.72%	64.75%	22.47%	66.67%
Latin/Central/South American	7.54%	5.72%	8.74%	1.32%	2.51%
Middle Eastern	1.51%	0.67%	1.09%	6.17%	2.51%
Pacific Islander	0.50%	0.34%	0.50%	0.00%	0.36%
South Asian	6.53%	7.41%	2.38%	14.10%	10.39%
Other	13.57%	3.37%	8.94%	1.76%	4.66%
No response (*n*)	15	1	5	0	1

#### Procedure

Volunteer participants were first presented with an on-screen consent document. After consenting to participate, measures were presented in random order with the exception of the “Who Am I?” prompt, which was presented first, and the demographic questionnaire, which was presented last. After completing all the measures, participants were presented with an on-screen debriefing form.

#### Measures

##### Identities

Participants’ central identities were measured as responses to the prompt: “Please write three answers to the question: “Who am I?” in the blanks. Answer as if you were giving the answers to yourself, not to somebody else. Try to provide your answers in single words or short phrases, if possible” (Grossack, 1960). These responses were piped into future questions.

##### Factors influencing concealability

Using open-ended prompts, participants provided one reason that each identity they generated would be easy to conceal as a response to the prompt: “It is easy to conceal that I am a [identity] because . . . ” Participants also provided one reason that it would be hard to conceal the same identities as responses to the prompt: “It is hard to conceal that I am a [identity] because . . . ” Participants were instructed to skip questions for which they could not generate responses.

##### Concealability beliefs

Participants answered a single item for each of the identities they generated: “In general, how easy or hard would it be to hide that you are a [identity] if you wanted to?” (*M* = 2.56, *SD* = 2.09). Responses were collected on 7-point scales anchored by response options (0) “Very hard” and (6) “Very easy,” scored such that higher scores indicate greater concealability. This item was included only for future exploratory work.

##### Demographics

Finally, participants completed a demographic questionnaire in which they reported their age, sex, gender identity, race, ethnic origin, multiracial status, sexual orientation, and how urban or rural their place of residence is.

### Analyses and Results

Statistical analyses for this and all subsequent studies were conducted in R version 3.5.1 (R Core Team, 2018).

#### Identities

Three research assistants coded participants’ identities into categories. Some responses included more than one category of identity (e.g., “White Woman” could be categorized into either “Race” or “Sex”). These were split into separate fields and coded individually. The result was 677 total identities (*M* = 3.16 identities per participant).

**Figure 1.**
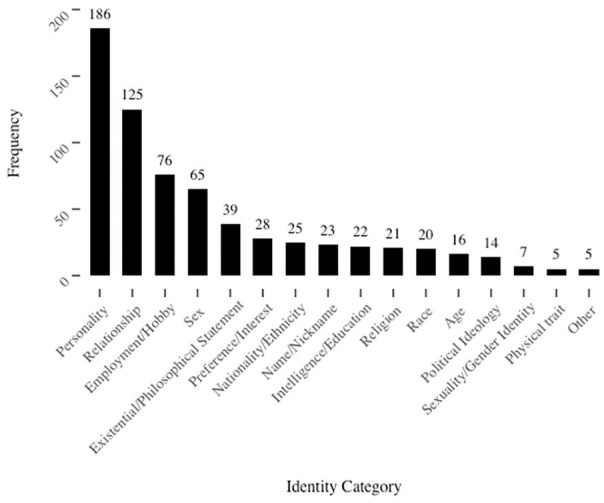
Frequency of identity categories in Study 1.

A preliminary list of categories was provided to the coders and they were encouraged to add new categories as they saw fit. Whenever a new category was added, it was added to coders’ lists and they revisited previously coded data to recode any responses they saw fit into the new category. All three coders coded the first 40% of responses, at which time coding was paused while Fleiss’ kappa was computed to assess inter-rater reliability. Inter-rater reliability was *κ* = .84, which was above the pre-registered threshold of *κ* = .75 (Fleiss et al., 2004). The first 40% of responses were coded into the category chosen by at least two of three coders and the first author broke three-way ties. Finally, the remaining 60% of responses were split between the coders and coded individually. The final list of categories and the frequency with which each was generated is included in Figure 1. Example responses for each category are included in the online supplement and all responses are available on the Open Science Framework.

#### Factors influencing concealability

Participants generated 369 reasons their identities were easy to conceal and 469 reasons that they were hard to conceal, totaling 838 reasons (*M* = 3.92 reasons per participant). This high response rate, despite participants having been instructed to leave responses blank if they could not think of a response, suggests that participants were able to generate responses with relative ease. The same coding procedure as that described for the identities was implemented here. The list of categories provided to coders was written with both “easy” and “hard” reasons in mind such that any category would be relevant to the ease and difficulty of concealment. After coding the first 40% of responses, inter-rater reliability for the “easy” reasons was *κ* = .72. Following a pre-registered plan than anticipated low inter-rater reliability, coders met in-person to resolve disputes. First, instances where coders had disagreed were identified. Then, each coder discussed their rationale for their decisions. Definitions and scopes of relevant categories and appropriate interpretations of the participant’s response were discussed until unanimous agreement was reached. The coders then coded the next 20% of “easy” responses independently and inter-rater reliability was calculated using only the newly-coded responses, yielding *κ* = .81. The remaining 40% of responses were divided between the coders and coded individually.

After coding the first 40% of responses to the “hard” concealability reasons, inter-rater reliability was *κ* = .46. The same process as that undertaken for the “easy” concealability reasons was undertaken three times, yielding *κ*s between .55 and .66, after which all coding was complete and disputes had been resolved.

**Figure 2.**
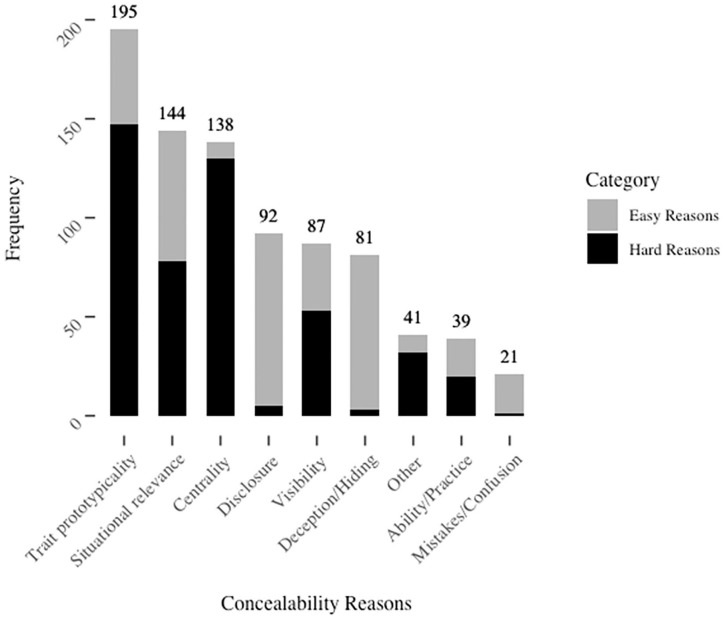
Frequency of categories of factors influencing the ease of concealment in Study 1.

The final list of categories generated was: “trait prototypicality” (someone’s representativity of their group), “situational relevance” (environmental factors that enable or inhibit concealment), “centrality” (how core the identity is to the person’s self-concept), “disclosure” (whether the identity had been previously disclosed to others), “visibility” (the identity’s ability to be perceived visually), “deception/hiding” (people’s comfort with or ability to lie about the identity or actively conceal it), “ability/practice” (people’s experience or skill with concealment), and “mistakes/confusion” (others’ knowledgeability about the group). Frequencies with which these were generated are shown in Figure 2. Example responses coded into each category are included in the online supplement and all responses are available on the Open Science Framework.

#### Candidate items

Twenty-nine candidate items were written with the goal of reflecting the concealment challenges and affordances that emerged from the data. Themes emerging from participants’ responses were used as inspiration for scale items. For example, responses about identity visibility inspired the item “How visible is the fact that you are [identity]?” Because each category of factor influencing the ease of concealability included both reasons that concealment would be easy and hard, the items were written to reflect both of these experiences.

To ensure the items would speak to factors relevant to a broad array of identities, we allowed participants to report on any type of identity, which resulted in a highly diverse set of identities. Furthermore, most items were inspired by themes that emerged from many participants’ responses concerning different types of identities, rather than any single response.

One additional item (Table 2, item 26) was added to reflect the previous finding that people hold a lay belief that their identities will inevitably “slip” out (Plaks et al., 2009). The complete list of items is included in Table 2.

**Table 2.** Candidate Scale Items and Loadings Onto a Single Factor.

Candidate item	Loading	Theme
How typical are you of an average [identity] person? (r)		Prototypicality
How surprised would most people be to learn that you are [identity]?		Prototypicality
How good an example of [identity] people are you? (r)		Prototypicality
How important a part of who you are is the fact that you are [identity]? (r)		Centrality
How affected are you by the fact that you are [identity]? (r)		Centrality
How much does being [identity] define who you are? (r)	.44	Centrality
How much do you like being [identity]? (r)		Centrality
Most of the time, how free do you feel to express the fact that you are [identity]? (r)		Situational Relevance
How often do you do things that make it obvious that you are [identity] to those around you? (r)	.68	Situational Relevance
How accepting are others of the fact that you are [identity]? (r)		Situational Relevance
How much does the fact that you are [identity] change from day to day?		Situational Relevance
How well do people tend to guess that you are [identity] even if you don’t tell them? (r)	.78	Disclosure
How often do people ask you if you are [identity]? (r)		Disclosure
How “out” do you consider yourself to be (as in, do people in your life know that you are [identity])? (r)	.42	Disclosure
How much would people believe you if you said you were not [identity]?		Deception/Hiding
How willing are you to alter things about yourself to prevent others from knowing that you are [identity]?		Deception/Hiding
How visible is the fact that you are [identity]? (r)	.80	Visibility
In general, how knowledgeable are people about what it means to be [identity]? (r)		Mistakes/Confusion
How frequently do people mix up [identity] people with a different type of person?		Mistakes/Confusion
How experienced are you at trying to hide the fact that you are [identity]?		Ability/Practice
How good are you at blending in, so that the fact that you are [identity] doesn’t stand out?		Ability/Practice
How able do you feel to act in a way that is the opposite of what people expect from people who are [identity]?		Ability/Practice
How easy is it for you to conceal that you are [identity]?	.44	Ability/Practice
How attentive are people to cues, signs, or signals that you are [identity]? (r)	.74	Other/General
How frequently do people notice that you are [identity]? (r)	.83	Other/General
How able do you feel to avoid “letting it slip” that you are [identity]?		Other/General
How quick are people to figure out that you are [identity]? (r)	.77	Other/General
How much does the fact that you are [identity] make you stand out? (r)	.52	Other/General
If you wanted to, how able would you be to stop being [identity]?		Other/General

*Note.* Loadings reflect EFA loadings for the one-factor solution presented in Study 2. Only loadings greater than .40 are shown. EFA = Exploratory Factor Analysis.

### Discussion

The present study used insights into the reasons that people believe an identity is easy or hard for them to conceal as the basis for scale item generation. The bottom-up approach taken in this study was adopted so that the items generated would reflect people’s experiences with the challenges of concealing. With the exception of one item added after analysis of the open-ended data, participants’ responses provided the basis for item development.

The wide range of responses participants generated, including themes such as previous disclosures, comfort with deception, identity centrality, and more, suggests that people’s subjective beliefs about the concealability of their own identities are not a simple function of visibility. Rather, a wide range of factors encompassing both practical and emotional considerations simultaneously influence people’s beliefs about their identities’ concealability.

## Study 2: Exploratory Factor Analysis

In Study 2, all 29 items were submitted to Exploratory Factor Analysis (EFA). Because each item asked about the concealability of a specific identity, participants first reported an identity they held. The identity was subsequently piped into questions to create idiographic items tailored to the participant. No specific set of factors was predicted to emerge.

### Method

#### Participants

Participants were recruited through Amazon Mechanical Turk (https://mturk.com; MTurk). Following guidelines reported in Hinkin (1995), our desired sample size was 10 participants per candidate item. In anticipation of exclusions, we oversampled by 20%, leading to a recruitment goal of 360 participants. In total, 361 participants were recruited. Missing data were dealt with using listwise deletion, leading to 23 exclusions. An additional 34 participants were excluded for failing at least one of the two attention checks. Finally, 6 participants who responded to the identity prompt with something other than an identity corresponding with the identity category they selected, thereby obscuring the meaning of future questions into which these responses were piped, were excluded. This left a final analytic dataset of 298 participants (see Table 1 for demographic details).

#### Procedure

The consent procedure was the same as Study 1. After consenting, participants completed a demographic questionnaire and a battery of survey measures including the 29 candidate items. At the end of the study, participants were thanked for their participation and received USD2.50.

#### Measures

##### Demographics

Participants completed a demographic questionnaire including the same items as Study 1, with the exception of race, which was not collected here, and religion, religiosity, political orientation, nationality, and country of residence, which were.

##### Identities

Participants were asked to choose an identity category they would like to be able to conceal. Participants were given the response options “age” (selected by *n* = 55), “ethnicity” (*n* = 9), “gender identity” (*n* = 3), “job” (*n* = 63), “nationality” (*n* = 9), “political ideology” (*n* = 84), “race” (*n* = 9), “religion” (*n* = 38), “sex” (*n* = 7), and “sexual orientation” (*n* = 21). These options were based on results from Study 1. Participants were then asked to provide their identity within the selected category. A participant who selected “religion” would therefore be asked: “What specific label or name would you use to describe your religion?”

##### Candidate scale items

The 29 candidate items (Table 2) were administered in random order on 5-point scales anchored by response options (0) “Not at all” and (4) “Extremely.” Eighteen of the 29 items were reverse-scored so that higher scores would correspond with greater concealability.

##### Attention checks

Two attention checks were administered. The first, administered in a random place within the candidate scale items, used the same response options as the scale items and the prompt: “For this question choose ‘slightly.’” Sixteen participants selected a response other than “slightly” and were excluded. The second attention check was administered at the end of the survey. Participants read the prompt: “At the start of the survey, you told us which one of your traits you most frequently wished you could conceal. Which trait was it?” and were shown the same set of 10 identity categories as they had been previously. Eighteen additional participants selected an incorrect identity and were excluded.

##### Additional measures

Detailed methods and results for additional variables are presented in Study 5.

### Data Preparation

All items and scales were tested for skew using the *psych* package in *R* (Revelle, 2018). Candidate scale item 11 had a skew greater than 1 and was therefore subjected to logarithmic transformation. In addition to being consistent with our pre-registered plan, we chose to transform this item and include it in the EFA rather than exclude it because it was written to assess an experience expressed by participants in Study 1 and we wished to have this represented.

### Analyses and Results

#### Exploratory factor analysis

EFA was performed using maximum likelihood factor analysis with promax rotation, which was selected because it does not assume orthogonality. Items with factor loadings above .40 on only one factor were retained. A summary of the EFA process is presented below. For full details on all explored solutions, see the online supplement (https://osf.io/pzcf9/).

**Figure 3.**
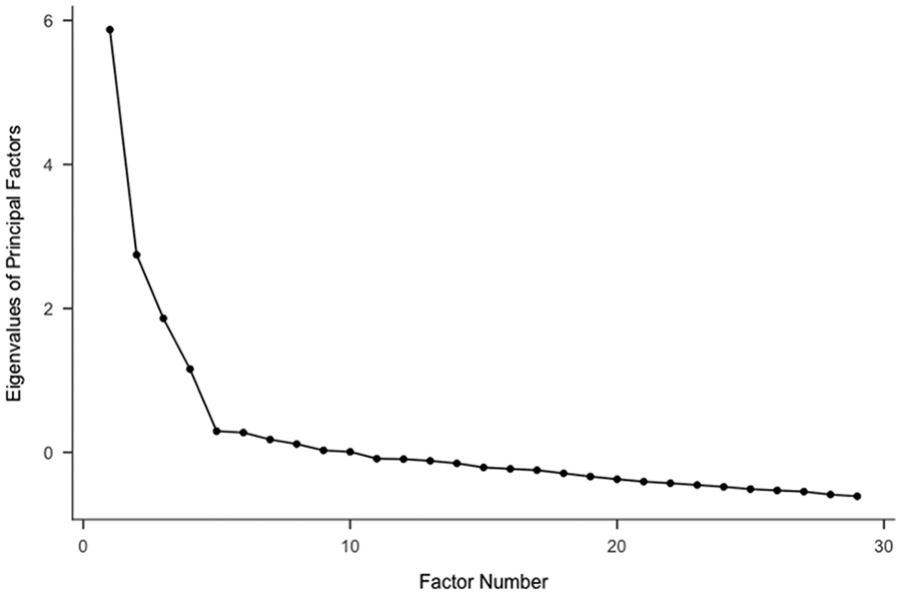
Scree plot from Study 2.

Following guidelines from Cattell (1966), inspection of a scree plot (Figure 3) led to the exploration of a four-factor solution. Only the first two factors had satisfactory internal reliability (total *ω* = .89; Factor 1 *ω* = .81; Factor 2 *ω* = .83; Factor 3 *ω* = .57; Factor 4 *ω* = .55), and item deletion could not improve reliability for Factors 3 and 4. Those were therefore dropped. Because there seemed to be no more than two coherent potential factors, a two-factor solution was explored next. Although both factors had good internal reliability (total *ω* = .85; Factor 1 *ω* = .87; Factor 2 *ω* = .75), they also had considerable cross-loadings, the removal of which substantially undermined Factor 2’s internal reliability.

Finally, a one-factor solution was assessed. Ten items loaded onto the single factor, whose composition largely resembled that of the first factors from both previous EFAs. The single factor achieved good internal reliability (*α* = .87). Because the four- and two-factor solutions each had significant psychometric challenges and because the one-factor solution was a parsimonious summary of themes that emerged from all three solutions, the one-factor solution was retained. Factor loadings for this solution are listed in Table 2.

### Discussion

The 10-item, 1-factor solution had strong psychometric properties and good face validity. Furthermore, it incorporated items from most (i.e., 7/9) of the themes that emerged from Study 1. That the single factor encompassed items written to reflect such a wide array of participants’ responses bolsters our confidence in proceeding with this single-factor solution. This solution was therefore retained as the preliminary scale pending Confirmatory Factor Analysis (CFA).

## Study 3: Confirmatory Factor Analysis

Study 3 sought to confirm the factor structure established in Study 2 and assess the scale’s invariance across focal identities.

### Method

#### Participants

Participants were recruited from Project Implicit. Sample size was determined following guidelines from Hinkin (1995) recommending at least 10 participants per parameter. This number was multiplied by five (i.e., the number of identities from which participants could choose to report) to increase the probability of having multiple identities with large enough sample sizes to assess measurement invariance. Therefore, the desired sample size was 1,050 participants. We planned to oversample by 20% to account for exclusions, leading to a recruitment goal of 1,260. However, because of higher than anticipated exclusions, we increased this goal by 350 participants prior to analysis to 1,610 participants.

Ultimately, 1,612 participants were recruited. Missing data were dealt with through listwise deletion, leading to 144 exclusions. Two hundred thirty additional participants failed at least one of two attention checks and were also excluded. Finally, 226 additional participants provided unusable answers to the identity prompt and were excluded. After these exclusions, a final analytic dataset of 1,012 participants remained (see Table 1 for demographic details).

#### Procedure

The consent and debriefing procedures for this study were the same as those used in Study 1. After consenting, participants completed a demographic questionnaire, two additional identity prompt questions, the 10 candidate scale items, and several additional survey measures. Participants in this study were volunteers.

#### Measures

##### Demographics

Participants answered the same items as in Study 1, with the exception of race, which was not asked about here, and religion, which was.

##### Identities

Identities were elicited using the same procedure as in Study 2. To increase cell sizes for individual categories and facilitate assessment of measurement invariance, participants were asked to choose from the five most frequently selected identities in Study 2: “age” (*n* = 221), “job” (*n* = 92), “political ideology” (*n* = 371), “religion” (*n* = 191), and “sexual orientation” (*n* = 137).

##### Preliminary scale items

The 10 preliminary scale items were administered in random order on 5-point scales anchored by response options (0) “Not at all” and (4) “Extremely.” All items except one were reverse-scored so that higher scores would correspond with greater concealability.

##### Attention checks

The same two attention checks as those administered in Study 2 were repeated here. The first was answered incorrectly by 194 participants who were removed. The second was answered incorrectly by 36 additional participants who were also excluded.

##### Additional measures

Additional variables are presented in Study 5.

### Data Preparation

All items and scales were tested for skew. No items had unacceptable skews so no transformations were applied.

### Analyses and Results

#### Confirmatory factor analysis

The 10-item preliminary Subjective Identity Concealability Scale was submitted to CFA using the lavaan package in *R* (Rosseel, 2012). This model achieved adequate fit, *SRMR* = .05, *CFI* = .94, *RMSEA* = .09, 90% CI [.08, .10] and good internal reliability, *α* = .89. However, in pursuit of more stringent model fit standards (i.e., *SRMR* < .08, *CFI* > .95, *RMSEA* < .07; Hooper et al., 2008), the two items most weakly loading onto the factor were dropped. These were: “How much does being [identity] define who you are?” and “How much does the fact that you are [identity] make you stand out?”

**Figure 4.**
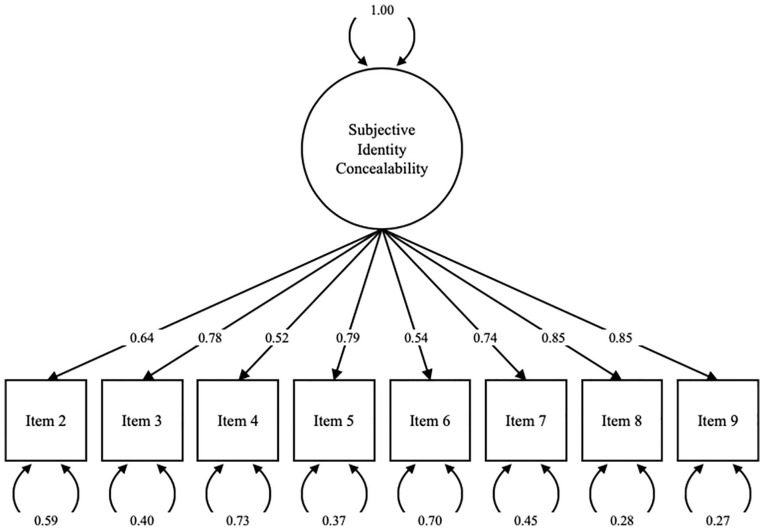
Final CFA model from Study 3. *Note.* CFA = Confirmatory Factor Analysis.

The remaining eight items were submitted to CFA, yielding strong fit, *SRMR* = .03, *CFI* = .98, *RMSEA* = .06, 90% CI [.05, .08]. As a robustness check, we cross-validated the model two ways. First, we re-ran the CFA in three random subsamples drawn from the CFA dataset of 210 participants each, sampled with replacement. Second, we re-ran the CFA in both other datasets collected up to this point that included the necessary variables (i.e., Study 2 dataset and an additional dataset reviewed in the online supplement). We sought converging evidence of strong model fit across each of these. Each re-analysis yielded strong fit (Subsample 1: *SRMR* = .03, *CFI* = .99, *RMSEA* = .04, 90% CI [.00, .08]; Subsample 2: *SRMR* = .03, *CFI* = .99, *RMSEA* = .06, 90% CI [.01, .09]; Subsample 3: *SRMR* = .03, *CFI* = .98, *RMSEA* = .06, 90% CI [.03, .09]; Study 2 sample: *SRMR* = .03, *CFI* = .98, *RMSEA* = .06, 90% CI [.03, .09]; online supplement sample: *SRMR* = .04, *CFI* = .96, *RMSEA* = .07, 90% CI [.04, .10]). Finally, internal reliability was calculated using the eight retained items, yielding *α* = .89. The eight-item scale was therefore taken as the final Subjective Identity Concealability Scale (see Appendix and Figure 4).

#### Measurement invariance

To ensure sufficiently large samples to assess measurement invariance, only identity categories chosen by 210 participants or more were included in the assessment of measurement invariance. Therefore, only age (*n* = 221) and political ideology (*n* = 371) were included. First, model fit was assessed within each category. Both yielded strong model fit (age: *SRMR* = .03, *CFI* = 1.00, *RMSEA* = .03, 90% CI [.00, .07]; political ideology: *SRMR* = .02, *CFI* = .99, *RMSEA* = .03, 90% CI [.00, .06]).

Configural invariance was then assessed with identity category as the grouping variable, which also yielded strong model fit, *SRMR* = .02, *CFI* = 1.00, *RMSEA* = .03, 90% CI [.00, .05]. To assess metric invariance, loadings were set to equal and the model was run again, yielding the following fit: *SRMR* = .09, *CFI* = .97, *RMSEA* = .07, 90% CI [.06, .09]. A difference score was then computed between configural CFI and metric CFI, yielding Δ*CFI* = .03. Because Δ*CFI* was greater than .01, our pre-registered threshold (Cheung & Rensvold, 2002), metric invariance was not achieved.

Next, partial metric invariance was assessed. Loadings for the one item that most strongly depressed metric CFI (“How often do you do things that make it obvious that you are [identity] to those around you?”) were set free and partial metric invariance was calculated, yielding the following fit: *SRMR* = .05, *CFI* = .99, *RMSEA* = .04, 90% CI [.02, .06], Δ*CFI* = .01. Therefore, partial metric invariance was achieved. Among the items for which partial metric invariance was achieved, partial scalar invariance was also investigated but was not achieved (Δ*CFI* > .01).

To assess the robustness of these results, the same analyses were run including the third most commonly chosen identity category: religion (*n* = 191). The same pattern of results emerged, yielding partial metric invariance with the same item set free.

### Discussion

In Study 3, an 8-item scale with strong model fit, strong internal reliability, and partial metric invariance was established. The factor structure established in Study 2 was largely confirmed, with slight alterations. The establishment of a coherent, internally reliable, and face-valid scale to measure the construct of subjective identity concealability enables the testing of the hypothesis of subjective identity concealability (see Study 5) and allows us to test its convergent and discriminant validity (see Study 4).

## Study 4: Scale Validation

The goal of Study 4 was to test the scale’s convergent and discriminant validity. We predicted that subjective identity concealability would correlate with constructs that represent factors people believe influence the ease of concealing. To select scales to tap such constructs, we looked to themes that emerged from Study 1, reasoning that a valid measure of participants’ beliefs about the concealability of an identity they hold should correlate with measures that reflect participants’ responses to Study 1’s open-ended prompts about factors that influence the easy of identity concealment. Subjective identity concealability was predicted not to correlate with scales that do not tap a theme emerging from Study 1. To assess validity, we therefore selected scales that may be considered conceptually similar to subjective identity concealability at face-value, had previously been found to correlate with other features of concealment, and other psychological constructs such as personality traits.

### Method

#### Participants

Participants were undergraduate psychology students at a Canadian university. In the absence of an anticipated effect size to use for power analysis, a recruitment goal of 250 participants was selected as the desired sample size following guidelines for stabilization of correlation estimates (Schönbrodt & Perugini, 2013), and we over-recruited to account for the likelihood of exclusions. In total, data were collected from 318 participants. Missing data were dealt with using listwise deletion, leading to 46 exclusions. Participants were excluded for providing unusable responses to the identity prompt, leading to 38 exclusions. Finally, 7 participants were excluded because of something they indicated in-person to a researcher (i.e., 5 participants indicated responding incorrectly to the focal identity prompt and 2 indicated a language barrier). This resulted in a final analytic sample of 227 participants (see Table 1 for demographic details).

#### Procedure

After being recruited through an institutional pool, participants were brought into a lab and presented with an on-screen consent document, which they were asked to read prior to consenting. Then, participants completed a demographic survey and a questionnaire wherein scales were presented in random order. Finally, participants were provided with a paper debrief document and asked to read it before leaving the lab. Participants were remunerated with partial course credit.

#### Measures

##### Demographics

Participants responded to the same items as Study 2 with the exception of country of residence, which was not measured here.

##### Identity

The same two items as those administered in Studies 2 and 3 were administered here. Participants chose from the same ten identity categories as in Study 2: age (*n* = 43), ethnicity (*n* = 33), gender identity (*n* = 7), job (*n* = 17), nationality (*n* = 22), political ideology (*n* = 41), race (*n* = 14), religion (*n* = 28), sex (*n* = 3), and sexual orientation (*n* = 19).

##### Subjective identity concealability

The 8-item Subjective Identity Concealability Scale (*α* = .88) was administered to participants on 5-point scales anchored by response options (0) “Not at all” and (4) “Extremely.” A mean of all items was taken after reverse-scoring all items but one so that higher scores indicate greater concealability (*M* = 2.01, *SD* = 0.98).

##### Dependent variables

Due to the large number of scales administered in this study, individual scales are reviewed in detail in the online supplement. Twelve scales were measured to assess convergent validity, 17 were administered to assess discriminant validity, and several additional measures were administered for hypothesis testing and are reviewed in Study 5.

### Data Preparation

All measures were tested for skew. Concealment behavior (skew = 1.33) and rejection sensitivity (skew = 1.09) displayed positive skews and were therefore subjected to logarithmic transformations.

### Analyses and Results

Because of the large number of tests conducted in this study, *p* values associated with each of the correlations were subjected to a false discovery rate correction using the method detailed in Benjamini and Yekuteli (2001). Raw *p* values are reported first, followed by adjusted *p* values, indicated by “*p_adj_.*”

#### Convergent validity

**Figure 5.**
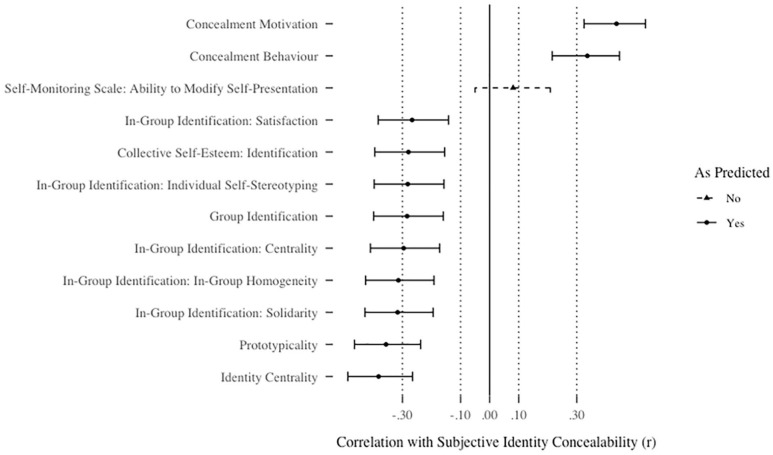
Correlations between subjective identity concealability and scales predicted to correlate with subjective identity concealability in Study 4. *Note.* Bars represent unadjusted 95% confidence intervals. Conclusions denoted in the legend reflect *p*-vales after correction for multiple tests.

The Subjective Identity Concealability Scale exhibited significant, small-to-medium correlations with 11 of the 12 scales predicted to converge with it (.27 ≤ *r*s ≤ .44, adjusted *p*s < .001). These correlations were all in the predicted directions. One scale did not correlate significantly with subjective identity concealability: the ability to modify self-presentation subscale of the Self-Monitoring Scale, *r*(225) = .08, *p* = .23, *p_adj_* = 1.00, 95% CI [−.05, .21]. For a summary of all correlations, see Figure 5.

#### Discriminant validity

**Figure 6.**
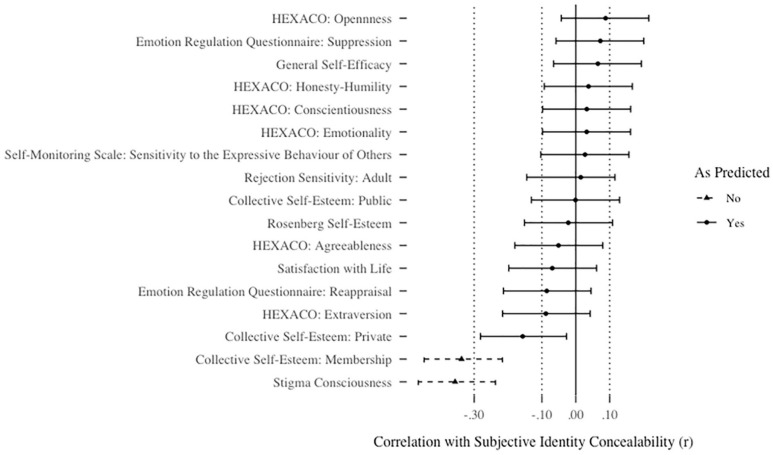
Correlations between subjective identity concealability and scales predicted not to correlate with subjective identity concealability in Study 4. *Note.* Bars represent adjusted 95% confidence intervals. Conclusions denoted in the legend reflect *p*-vales after correction for multiple tests.

In 15 of 17 cases, significant correlations were not found with the scales with which divergence was predicted (all *r*s ≤ .16, all adjusted *p*s > .05). The two scales that did correlate with the Subjective Identity Concealability Scale were the membership subscale of the Collective Self-Esteem Scale, *r*(225) = −.34, *p* < .001, *p_adj_ <* .001, 95% CI [−.45, −.22], and stigma consciousness, *r*(225) = −.36, *p* < .001, *p_adj_ <* .001, 95% CI [−.46, −.24]. For a summary of all correlations, see Figure 6.

### Discussion

Pre-registered, directional predictions for the scales with which subjective identity concealability should and should not correlate were supported in nearly all cases. This pattern of results suggests that the Subjective Identity Concealability Scale is a valid measure of factors people feel influence the ease or difficulty of concealing an identity and that it is not redundant with other constructs currently in the literature.

Although contrary to our pre-registered prediction, the negative relationship between subjective identity concealability and stigma consciousness raises an intriguing mechanistic possibility. This prediction was originally made consistent with how all the predictions concerning convergence and divergence were made (i.e., convergence was only sought with scales reflecting themes that emerged in Study 1). Stigma consciousness did not fit this criterion. Nonetheless, the hypothesis of subjective identity concealability, to be tested in Study 5, states that people who believe an identity is concealable may face lower levels of the consequences of fearing identity-based judgment. To the extent that this is true, one way in which it may manifest is that people who believe an identity is concealable are less chronically burdened by—and therefore less chronically conscious of—their stigmatized group membership. If so, reductions in stigma consciousness may mediate the relationship between subjective identity concealability and the consequences of fearing identity-based judgment. Future research should test this possibility.

## Study 5: Hypothesis Tests

The aim of Study 5 was to test the hypothesis that people who believe an identity they hold is concealable are less burdened by the costs of fearing identity-based judgment. Given that people differ in the degree to which they feel able to conceal an identity, their experiences with people from different groups should likewise differ such that the extent to which someone expects negative outcomes related to an intergroup interaction should be inversely related to the extent to which they believe them-self to be in control over the outgroup member’s knowledge of the relevant identity. This hypothesis is operationalized with four dependent variables: threat of being stereotyped, experiences of being the target of prejudice, intergroup anxiety, and situational avoidance,^2^ measures of which were administered in studies throughout the present paper. These relationships are meta-analyzed and presented together in the present study, following guidelines from Goh et al. (2016).

We additionally assessed subjective identity concealability’s associations with ingroup attitudes and authenticity. Although we did not have specific *a priori* hypotheses for these tests, we nonetheless wished to explore these potentially negative associations of subjective identity concealability.

### Method

Random effects meta-analyses were conducted using the *metafor* package in *R* (Viechtbauer, 2010). Data were drawn from four studies: Studies 2–4 of the present paper and one additional study which is reviewed in detail in the online supplement.

#### Samples

Demographic information for all five studies is summarized in Table 2 (see the columns for Studies 2–4 and “Supp.”). Together, they draw on data from 1,817 participants.

#### Measures

##### Subjective identity concealability

The Subjective Identity Concealability Scale was measured in all samples. Items were posed on 5-point scales anchored by (0) “Not at all” and (4) “Extremely,” scored such that higher scores indicate greater belief in the concealability of one’s identity, and the scale had good internal reliability (Supplement sample *M* = 2.56, *SD* = 0.79, *α* = .84; Study 2 *M* = 2.31, *SD* = 0.84, *α* = .87; Study 3 *M* = 2.39, *SD* = 0.89, *α* = .89; Study 4 *M* = 2.01, *SD* = 0.98, *α* = .88).

##### Threat of being stereotyped

A single-item measure of threat of being stereotyped adapted from Cohen and Garcia (2005) was administered: “I worry that people will draw conclusions about me, based on what they think about [identity] people.” The item was posed on a 7-point scale anchored by (−3) “Strongly Disagree” and (3) “Strongly Agree” where higher scores indicate greater threat. This measure was administered in four samples (Supplement sample: *M* = −0.66, *SD* = 2.03; Study 2 *M* = 0.84, *SD* = 1.69; Study 3 *M* = 0.75, *SD* = 1.88; Study 4 *M* = 0.89, *SD* = 1.72).

##### Experience of prejudice

Participants responded to the prompt “I experience prejudice because I am [identity]” on a 7-point scale anchored by (−3) “Strongly Disagree” and (3) “Strongly Agree” scored such that higher scores indicate greater experience of prejudice. This measure was administered in four samples (Supplement sample: *M* = −1.21, *SD* = 1.92; Study 2 *M* = −0.06, *SD* = 1.79; Study 3 *M* = −0.09, *SD* = 1.84; Study 4 *M* = −0.09, *SD* = 1.76).

##### Situational avoidance

Situational avoidance was measured using three items: “Because I am [identity], I sometimes avoid doing things I would otherwise like to do,” “There are things in my life that I would be more comfortable doing if I were not [identity],” and “There are things in my life that I would spend more time doing if I were not [identity].” Items were posed on 7-point scales anchored by (−3) “Strongly Disagree” and (3) “Strongly Agree” and scores were calculated by taking a mean of the three items such that higher scores indicate greater proclivity to avoid otherwise-desirable activities. Situational avoidance was assessed in two samples (Study 3 *M* = −0.52, *SD* = 1.71, *α* = .82; Study 4 *M* = −0.08, *SD* = 1.66, *α* = .78).

##### Intergroup anxiety

Intergroup anxiety was measured using 10 items adapted from Stephan and Stephan (1985). This measure posed the same question 10 times

(If you were interacting with a group of people (e.g., talking with them, working on a project with them, etc.) and you were the only [identity] person in the group, how would you feel compared to occasions when you are interacting with other people who are [identity]?)

and asked participants to respond with how intensely they would feel a different emotion for each question (e.g., awkward, self-conscious, irritated, etc.) on 5-point scales anchored by (0) “Not at all” and (4) “Extremely.” Scores were calculated as a mean of the 10 items after reverse-scoring three items so that higher scores correspond with greater anxiety. Intergroup anxiety was assessed in two samples (Study 3 *M* = 1.35, *SD* = 0.69, *α* = .85; Study 4 *M* = 1.37, *SD* = 0.74, *α* = .85).

##### Ingroup attitudes

A feeling thermometer assessing participants’ attitudes toward their ingroup was administered. The item “Please rate how warm or cold you feel toward the following group: [identity] people” was posed on a 7-point scale anchored by response options (−3) “Very Cold” and (3) “Very Warm,” scored such that higher scores indicate warmer feelings toward the participant’s ingroup. Ingroup attitudes were measured in four samples (Supplement sample: *M* = 1.35, *SD* = 1.35; Study 2 *M* = 1.44, *SD* = 1.51; Study 3 *M* = 1.73, *SD* = 1.22; Study 4 *M* = 1.34, *SD* = 1.31).

##### Authenticity

The three-factor Authenticity Scale (Wood et al., 2008) was administered in the Study 4 sample only. The scale is composed of three four-item subscales: authentic living (example item: “I think it is better to be yourself, than to be popular.”; *M* = 4.53, *SD* = 0.98, α = .76), accepting external influence (example item: “I am strongly influenced by the opinions of others.”; *M* = 3.20, *SD* = 1.41, *α* = .86), and self-alienation (example item: “I don’t know how I really feel inside.”; *M* = 2.60, *SD* = 1.47, *α* = .81). Questions were posed on 7-point scales anchored by (0) “Does not describe me at all” and (6) “Describes me very well.” Each subscale’s score was calculated by taking a mean of its items such that higher scores indicate higher levels of the experience assessed by the subscale.

### Analyses and Results

#### Confirmatory meta-analytic tests

**Figure 7.**
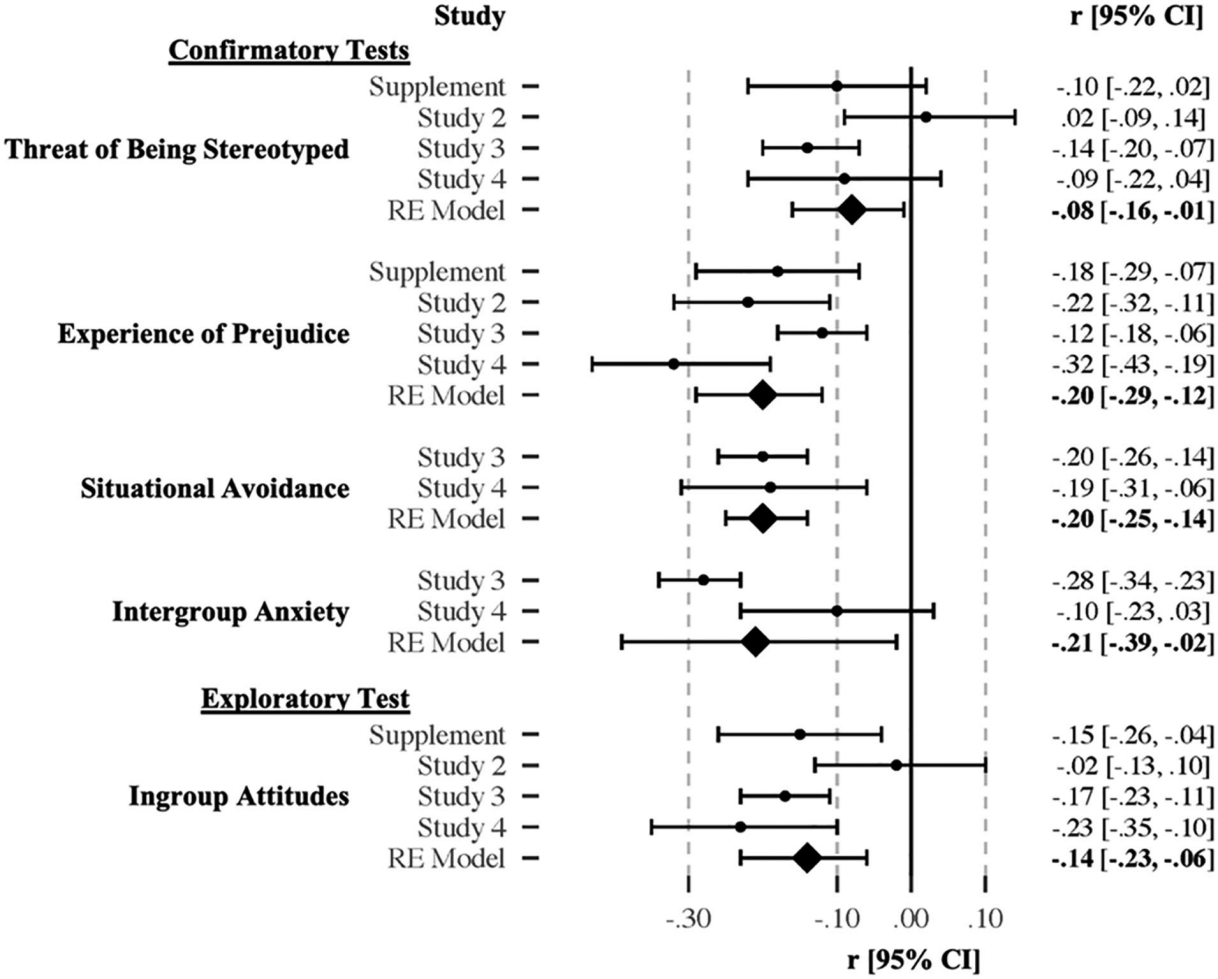
Forest plot depicting associations with subjective identity concealability in Study 5.

A forest plot visualizing all results is included as Figure 7.

##### Threat of being stereotyped

Across four studies (*n* = 1,817), participants high in subjective identity concealability reported lower levels of threat of being stereotyped, *r* = −.08, 95% CI [−.16, −.01], *z* = −2.32, *p* = .02.

##### Experience of prejudice

Across the same four studies (*n* = 1,817), participants higher on subjective identity concealabilityreported experiencing less prejudice on the basis of their focal identity, *r* = −.20, 95% CI [−.29, −.12], *z* = −4.64, *p* < .001.

##### Situational avoidance

Across two studies (*n* = 1,239), participants higher in subjective identity concealability reported being less prone to avoiding otherwise-desirable activities on account of their focal identity, *r* = −.20, 95% CI [−.25, −.14], *z* = −6.97, *p* < .001.

##### Intergroup anxiety

In two studies (*n* = 1,239), people higher in subjective identity concealability were lower in intergroup anxiety, *r* = −.21, 95% CI [−.39, −.02], *z* = −2.19, *p* = .03.

##### Exploratory meta-analytic test of ingroup attitudes

Across all four studies (*n* = 1,817), people who believed an identity was more concealable also had colder feelings toward their ingroup, *r* = −.14, 95% CI [−.23, −.06], *z* = −3.41, *p* < .001.

##### Additional exploratory tests

Subjective identity concealability did not correlate with any of the three factors of authenticity: authentic living, *r*(225) = −.04, *p* = .58, 95% CI [−.17, .09], accepting external influence, *r*(225) = .06, *p* = .38, 95% CI [−.07, .19], and self-alienation, *r*(225) = .01, *p* = .83, 95% CI [−.12, .14].

### Discussion

Consistent with our hypothesis, subjective identity concealability was consistently related to lower levels of the psychological costs of fearing identity-based judgment across all four dependent variables.

It is worth noting that people who believed an identity was more concealable held colder attitudes toward members of their ingroup. This is perhaps unsurprising given the qualitative data collected in Study 1; factors related to identity centrality were the second most common category of response from participants to the question of what made concealing harder for them to do, suggesting that people for whom an identity is particularly important may see its concealment as more difficult. Bolstering this reasoning, in Study 3, subjective identity concealability correlated negatively with several operationalizations of centrality and identification.

Finally, because of the well-known association between concealment behavior and feelings of inauthenticity (Newheiser & Barreto, 2014; Riggle et al., 2017), we additionally assessed the association between subjective identity concealability and authenticity. *Beliefs* about concealability should not impact feelings of authenticity in the same way as actual concealment behavior. Results of the present study support this reasoning; subjective identity concealability did not correlate with authenticity.

## General Discussion

Across four pre-registered studies and a set of internal meta-analyses, we explored subjective identity concealability, developed and validated a tool to measure it, and tested, and found support for, a set of theory-based hypotheses.

Subjective identity concealability does not concern only beliefs about identity visibility, nor does it concern only beliefs about one’s own skillfulness at concealment. Rather, it concerns many factors people feel influence the ease or difficulty of concealment simultaneously, including visibility, prior disclosures, skillfulness at concealing, and even factors external to the self. This amalgam reflects a multitude of factors people feel influence the ease or difficulty of concealment.

### Subjective Identity Concealability is Associated With Theoretically Predicted Outcomes

Subjective identity concealability is associated with lower levels of the costs of fearing intergroup judgment such as identity threat and intergroup anxiety. These results indicate that, for people concerned about being judged negatively on the basis of an identity, a sense that the identity is chronically on-display is a threatening experience that may be attenuated by a belief that they are in control over who knows that they hold the identity.

The real-world consequences of fearing identity-based judgment extend to domains including education, the workplace, and intergroup relations (see Spencer et al., 2016; Stephan, 2014 for reviews). Little research to date has examined the extent to which feeling in control of an identity’s concealment or disclosure is related to these outcomes. By identifying an individual difference predictor of these important outcomes, the present work sheds light on these processes. Furthermore, follow-up work has already begun to demonstrate that the correlates of subjective identity concealability extend to these domains; people who believe an identity is concealable report greater comfort initiating intergroup interactions and have more and higher quality intergroup contact (Le Forestier et al., 2020).

### Undesirable Correlates of Subjective Identity Concealability

Although we have documented a set of desirable correlates of subjective identity concealability in the present work, the experience of believing an identity is concealable may also have negative correlates, particularly in domains known to be associated with concealment behavior (e.g., health, authenticity, relational quality, internalized prejudice, etc.). Thus far, there is mixed evidence on this topic. In Study 5 of the present paper, subjective identity concealability was not found to correlate with authenticity. Similarly, in follow-up work, subjective identity concealability has been associated with *higher* quality interpersonal relationships (in intergroup contexts), rather than lower (Le Forestier et al., 2020). However, in Study 5 of the present work, subjective identity concealability correlated negatively with attitudes toward one’s ingroup, and with multiple measures of ingroup identification in Study 4. Other potential negative outcomes remain as-of-yet unexplored.

Why might subjective identity concealability be associated with some of the same negative experiences as concealment behavior but not all? Some of these experiences are likely involved in the development of subjective identity concealability. For example, lower identity centrality emerged in Study 1’s data as a factor that rendered concealment easier. It is therefore unsurprising that measures of identification and ingroup attitudes correlated negatively with subjective identity concealability. However, in the absence of a proposed mechanism similar to that which motivates our primary hypotheses, other negative experiences related to concealment but that do not contribute to the development of subjective identity concealability may be less likely to be associated with subjective identity concealability. This is because believing an identity is concealable alone may not be sufficient to engender such outcomes. This account is informed by findings throughout the present paper and is consistent with findings from elsewhere in the concealment literature, such as the finding that more harm is done by active engagement in concealment behavior than by more passive forms of concealment (Quinn et al., 2017).

### Strengths, Limitations, and Future Directions

A strength of this work is that it is not limited to a single identity or domain. That effects were found across a wide array of identities demonstrates the generality of the effect. Furthermore, subsequent work has found that effects of subjective identity concealability hold both collapsing across identities and within individual identities, demonstrating the independence of the present effect from group membership alone (Le Forestier et al., 2020).

However, given that stigma underlies the motivation for the present work, a limitation is that these data cannot speak specifically to the experiences of those with traditionally stigmatized identities. To avoid assuming which identities participants would feel stigmatized on the basis of, we elected not to screen for participants with a set of pre-determined identities. Rather, in Studies 2–4, we asked participants to report on an identity they sometimes wished to conceal, which would imply that they felt burdened by others’ appraisals of that identity. This, of course, is an imperfect proxy for stigma, as there may be reasons other than stigma that someone would want to conceal an identity.

An additional strength is that our data pull from several different sources, including online volunteers from Project Implicit, paid online participants on MTurk, and undergraduate students from an institutional subject pool. Our ability to synthesize across them, particularly in Study 5, rather than relying exclusively on any one of them, renders less likely the possibility that idiosyncrasies of one particular subject pool account for the present results. At the same time, none of these samples is entirely representative of the general population. Future work should continue sampling participants from diverse sources to test the generalizability of the present findings.

Given our causal hypothesis that subjective identity concealability protects people against the negative outcomes of stigma, a limitation of the current work is that the method employed is correlational. Future research should test whether the theoretically-predicted relationship between subjective identity concealability and the costs of fearing intergroup judgment is causal in nature.

Additional future work is needed to explore the conceptual space of concealability beliefs. In the present work, we define subjective identity concealability as a person’s belief of whether attempted concealment of an identity they hold is likely to be successful. We have not distinguished between beliefs rooted in the person’s sense of how concealable an identity is in general (e.g., how concealable any person’s sexual orientation is) and beliefs rooted in the person’s sense of self-efficacy (e.g., how good the person, them-self, is at concealing a specific identity). While subjective identity concealability as a construct, and the scale we have developed to assess it, does not distinguish between these, future work should assess each of these sources of variance, in addition to others including one’s environment and one’s life events as they unfold. For example, in instances where one’s beliefs about concealability do not reflect actual success at concealment, whether, how, and under what conditions someone would update their concealability beliefs is unknown and should be studied.

Finally, future work should explore the negative correlates of subjective identity concealability in greater depth. This includes potential consequences to the self of distancing from one’s ingroup in this way, long-term relational effects of concealability beliefs, and the extent to which the relationship between subjective identity concealability and concealment behavior is involved in these outcomes.

### Conclusion

The present studies explore the construct of subjective identity concealability and demonstrate its association with important psychological outcomes. This sheds light on a previously overlooked aspect of people’s experiences in intergroup contexts as they are experienced by those with identity-based judgment concerns. The present research demonstrates that people’s beliefs about the concealability of their own identities are related to desirable outcomes in intergroup contexts such as reduced identity threat and intergroup anxiety.

## Supplemental Material

sj-docx-1-psp-10.1177_01461672211010038 – Supplemental material for Subjective Identity Concealability and the Consequences of Fearing Identity-Based Judgment Supplemental material, sj-docx-1-psp-10.1177_01461672211010038 for Subjective Identity Concealability and the Consequences of Fearing Identity-Based Judgment by Joel M. Le Forestier, Elizabeth Page-Gould, Calvin K. Lai and Alison L. Chasteen in Personality and Social Psychology Bulletin

## Appendix

### Subjective Identity Concealability Scale

Please take a moment to consider your identity as a [identity] person. Think about how the fact that you are [identity] affects you. Think about what it is like to be [identity]. Then, answer the following questions:

1. How often do you do things that make it obvious that you are [identity] to those around you? (Reverse-scored)
2. How well do people tend to guess that you are [identity] even if you don’t tell them? (Reverse-scored)
3. How “out” do you consider yourself to be (as in, do people in your life know that you are [identity])? (Reverse-scored)
4. How visible is the fact that you are [identity]? (Reverse-scored)
5. How easy is it for you to conceal that you are [identity]?
6. How attentive are people to cues, signs, or signals that you are [identity]? (Reverse-scored)
7. How frequently do people notice that you are [identity]? (Reverse-scored)
8. How quick are people to figure out that you are [identity]? (Reverse-scored)

**Table:**

0	1	2	3	4
Not at all	Slightly	Moderately	Quite a bit	Extremely

## Notes

1 “Supp.” refers to one additional study presented in the online supplement. Data from this study are included in the internal meta-analyses presented in Study 5. See Study 5 and the online supplement for further detail: https://osf.io/pzcf9/.

2 Belonging uncertainty was also assessed but the measure failed to achieve adequate internal reliability in either sample. See the online supplement for details.

## References

[bibr1-01461672211010038] Beals K. P. Peplau L. A. Gable S. L. (2009). Stigma management and well-being: The role of perceived social support, emotional processing, and suppression. Personality and Social Psychology Bulletin, 35(7), 867–879. 10.1177/0146167209334783 19403792

[bibr2-01461672211010038] Becker H. (1963). Outsiders: Studies in the sociology of deviance. The Free Press.

[bibr3-01461672211010038] Benjamini Y. Yekuteli D. (2001). The control of the false discovery rate in multiple testing under dependency. The Annals of Statistics, 29(4), 1165–1188.

[bibr4-01461672211010038] Butz D. A. Plant E. A. (2006). Perceiving outgroup members as unresponsive: Implications for approach-related emotions, intentions, and behavior. Journal of Personality and Social Psychology, 91(6), 1066–1079. 10.1037/0022-3514.91.6.1066 17144765

[bibr5-01461672211010038] Cattell R. B. (1966). The scree test for the number of factors. Multivariate Behavioral Research, 1(2), 245–276. 10.1207/s15327906mbr0102 26828106

[bibr6-01461672211010038] Chaudoir S. Fisher J. D. (2010). The disclosure process model: Understanding disclosure decision-making and post-disclosure outcomes among people living with a concealable stigmatized identity. Psychological Bulletin, 136(2), 236–256. 10.1037/a0018193.The 20192562 PMC2922991

[bibr7-01461672211010038] Cheung G. W. Rensvold R. B. (2002). Evaluating goodness-of-fit Indexes for testing measurement invariance. Structural Equation Modeling: A Multidisciplinary Journal, 9(2), 233–255.

[bibr8-01461672211010038] Cohen G. L. Garcia J. (2005). “I am us”: Negative stereotypes as collective threats. Journal of Personality and Social Psychology, 89(4), 566–582. 10.1037/0022-3514.89.4.566 16287419

[bibr9-01461672211010038] Cole E. R. Yip T. (2008). Using outgroup comfort to predict black students’ college experiences. Cultural Diversity & Ethnic Minority Psychology, 14(1), 57–66. 10.1037/1099-9809.14.1.57 18230001

[bibr10-01461672211010038] Cole S. W. Kemeny M. E. Taylor S. E. Visscher B. R. (1996). Elevated physical health risk among gay men who conceal their homosexual identity. Health Psychology, 15(4), 243–251. 10.1037/0278-6133.15.4.243 8818670

[bibr11-01461672211010038] Gaddis M. (2015). Discrimination in the credential society: An audit study of race and college selectivity in the labor market. Social Forces, 93(4), 1451–1459. 10.1093/sf/sou111

[bibr12-01461672211010038] Goffman E. (1963). Stigma: Notes on the management of spoiled identity. Prentice-Hall.

[bibr13-01461672211010038] Goh J. X. Hall J. A. Rosenthal R. (2016). Mini meta-analysis of your own studies: Some arguments on why and a primer on how. Social & Personality Psychology Compass, 10(10), 535–549. 10.1111/spc3.12267

[bibr14-01461672211010038] Goh J. X. Kort D. N. Thurston A. M. Benson L. R. Kaiser C. R. (2019). Does concealing a sexual minority identity prevent exposure to prejudice? Social Psychological & Personality Science, 10, 1056–1064. 10.1177/1948550619829065

[bibr15-01461672211010038] Grossack M. M. (1960). The “who am I” test. Journal of Social Psychology, 51(2), 399–402. 10.1080/00224545.1960.9922048

[bibr16-01461672211010038] Hinkin T. R. (1995). A review of scale development practices in the study of organizations. Journal of Management, 21(5), 967–988.

[bibr17-01461672211010038] Hooper D. Coughlan J. Mullen M. (2008). Structural equation modelling: Guidelines for determining model fit. Electronic Journal of Business Research Methods, 6(1), 63–60.

[bibr18-01461672211010038] Jackson S. D. Mohr J. J. (2016). Conceptualizing the closet: Differentiating stigma concealment and nondisclosure processes. Psychology of Sexual Orientation and Gender Diversity, 3(1), 80–92.

[bibr19-01461672211010038] Kang S. K. DeCelles K. A. Tilcsik A. Jun S. (2016). Whitened résumés: Race and self-presentation in the labor market. Administrative Science Quarterly, 61(3), 469–502. 10.1177/0001839216639577

[bibr20-01461672211010038] Le Forestier J. M. Page-Gould E. Lai C. K. Chasteen A. L . (2020). Concealability beliefs facilitate navigating intergroup contexts. European Journal of Social Psychology, 50, 1210–1226. 10.1002/ejsp.2681

[bibr21-01461672211010038] Levy S. R. Chiu C. Hong Y. (2006). Lay theories and intergroup relations. Group Processes & Intergroup Relations, 9(1), 5–24. 10.1177/1368430206059855 PMC3832063 24260013

[bibr22-01461672211010038] Levy S. R. Plaks J. E. Hong Y. Y. Chiu C. Y. Dweck C. S. (2001). Static versus dynamic theories and the perception of groups: Different routes to different destinations. Personality and Social Psychology Review, 5(2), 156–168. 10.1207/S15327957PSPR0502_6

[bibr23-01461672211010038] Major B. Gramzow R. H. (1999). Abortion as stigma: Cognitive and emotional implications of concealment. Journal of Personality and Social Psychology, 77(4), 735–745. 10.1037/0022-3514.77.4.735 10531670

[bibr24-01461672211010038] Mallett R. K. Wilson T. D. Gilbert D. T. (2008). Expect the unexpected: Failure to anticipate similarities leads to an intergroup forecasting error. Journal of Personality and Social Psychology, 94(2), 265–277. 10.1037/0022-3514.94.2.94.2.265 18211176

[bibr25-01461672211010038] Mohr J. J. Daly C. A. (2008). Sexual minority stress and changes in relationship quality in same-sex couples. Journal of Social and Personal Relationships, 25(6), 989–1007. 10.1177/0265407508100311

[bibr26-01461672211010038] Mohr J. J. Fassinger R. (2000). Measuring dimensions of lesbian and gay male experience. Measurement and Evaluations in Counseling and Development, 33, 66–90.

[bibr27-01461672211010038] Newheiser A.-K. Barreto M. (2014). Hidden costs of hiding stigma: Ironic interpersonal consequences of concealing a stigmatized identity in social interactions. Journal of Experimental Social Psychology, 52, 58–70. 10.1016/j.jesp.2014.01.002

[bibr28-01461672211010038] Pachankis J. E. Bränström R. (2018). Hidden from happiness: Structural stigma, sexual orientation concealment, and life satisfaction across 28 countries. Journal of Consulting and Clinical Psychology, 86(5), 403–415. 10.1037/ccp0000299 29683698

[bibr29-01461672211010038] Pachankis J. E. Hatzenbuehler M. L. Wang K. Burton C. L. Crawford F. W. Phelan J. C. Link B. G. (2018). The burden of stigma on health and well-being: A taxonomy of concealment, course, disruptiveness, aesthetics, origin, and peril across 93 stigmas. Personality and Social Psychology Bulletin, 44(4), 451–474. 10.1177/0146167217741313 29290150 PMC5837924

[bibr30-01461672211010038] Pachankis J. E. Mahon C. P. Jackson S. D. Fetzner B. K. Bränström R. (2020). Sexual orientation concealment and mental health: A conceptual and meta-analytic review. Psychological Bulletin, 146(10), 831–871. 10.1037/bul0000271 32700941 PMC8011357

[bibr31-01461672211010038] Plaks J. E. McNichols N. K. Fortune J. L. (2009). Thoughts versus deeds: Distal and proximal intent in lay judgments of moral responsibility. Personality and Social Psychology Bulletin, 35(12), 1687–1701. 10.1177/0146167209345529 19726810

[bibr32-01461672211010038] Quinn D. M. Weisz B. M. Lawner E. K. (2017). Impact of active concealment of stigmatized identities on physical and psychological quality of life. Social Science & Medicine, 192, 14–17. 28941787 10.1016/j.socscimed.2017.09.024

[bibr33-01461672211010038] R Core Team. (2018). R: A language and environment for statistical computing.

[bibr34-01461672211010038] Revelle W. (2018). psych: Procedures for personality and psychological research. Northwestern University.

[bibr35-01461672211010038] Richeson J. A. Trawalter S. (2005). Why do interracial interactions impair executive function? A resource depletion account. Journal of Personality and Social Psychology, 88(6), 934–947. 10.1037/0022-3514.88.6.934 15982114

[bibr36-01461672211010038] Riggle E. D. B. Rostosky S. S. Black W. W. Rosenkrantz D. E. (2017). Outness, concealment, and authenticity: Associations with LGB individuals’ psychological distress and well-being. Psychology of Sexual Orientation and Gender Diversity, 4(1), 54–62.

[bibr37-01461672211010038] Rosseel Y. (2012). lavaan: An R package for structural equation modeling. Journal of Statistical Software, 48(2), 1–36.

[bibr38-01461672211010038] Sanchez D. T. Bonam C. M. (2009). To disclose or not to disclose biracial identity: The effect of biracial disclosure on perceiver evaluations and target responses. Journal of Social Issues, 65(1), 129–149. 10.1111/j.1540-4560.2008.01591.x

[bibr39-01461672211010038] Schönbrodt F. D. Perugini M. (2013). At what sample size do correlations stabilize? Journal of Research in Personality, 47, 609–612. 10.1016/j.jrp.2013.05.009

[bibr40-01461672211010038] Spencer S. J. Logel C. Davies P. G. (2016). Stereotype threat. Annual Review of Psychology, 67, 415–437. 10.1146/annurev-psych-073115-103235 26361054

[bibr41-01461672211010038] Steele C. M. Aronson J. (1995). Stereotype threat and the intellectual test performance of African Americans. Journal of Personality and Social Psychology, 797–811. 7473032 10.1037//0022-3514.69.5.797

[bibr42-01461672211010038] Steele C. M. Spencer S. J. Aronson J. (2002). Contending with group image: The psychology of stereotype and social identity threat. Advances in Experimental Social Psychology, 34, 379–440. 10.1016/S0065-2601(02)80009-0

[bibr43-01461672211010038] Stephan W. G. (2014). Intergroup anxiety: Theory, research, and practice. Personality and Social Psychology Review, 18(3), 239–255. 10.1177/1088868314530518 24815215

[bibr44-01461672211010038] Stephan W. G. Stephan C. W. (1985). Intergroup anxiety. Journal of Social Issues, 41(3), 157–175. 10.1111/j.1540-4560.1985.tb01134.x

[bibr45-01461672211010038] Trawalter S. Adam E. K. Chase-Lansdale P. L. Richeson J. A. (2012). Concerns about appearing prejudiced get under the skin: Stress responses to interracial contact in the moment and across time. Journal of Experimental Social Psychology, 48(3), 682–693. 10.1016/j.jesp.2011.12.003 22711918 PMC3375720

[bibr46-01461672211010038] Ullrich P. M. Lutgendorf S. K. Stapleton J. T. (2003). Concealment of homosexual identity, social support and CD4 cell count among HIV-seropositive gay men. Journal of Psychosomatic Research, 54(3), 205–212. 10.1016/S0022-3999(02)00481-6 12614830

[bibr47-01461672211010038] Ulrey K. L. Amason P. (2001). Intercultural communication between patients and health care providers: An exploration of intercultural communication effectiveness, cultural sensitivity, stress, and anxiety. Health Communication, 13(4), 449–463. 10.1207/S15327027HC1304_06 11771806

[bibr48-01461672211010038] Van Zomeren M. Fischer A. H. Spears R . (2007). Testing the limits of tolerance: How intergroup anxiety amplifies negative and offensive responses to out-group-initiated contact. Personality and Social Psychology Bulletin, 33(12), 1686–1699. 10.1177/0146167207307485 18000103

[bibr49-01461672211010038] Viechtbauer W. (2010). Conducting meta-analyses in R with the metafor package. Journal of Statistical Software, 36(3), 1–48.

[bibr50-01461672211010038] Weisz B. M. Quinn D. M. Williams M. K. (2016). Out and healthy: Being more “out” about a concealable stigmatized identity may boost the health benefits of social support. Journal of Health Psychology, 21(12), 2934–2943. 10.1177/1359105315589392 26078297

[bibr51-01461672211010038] Wood A. M. Linley P. A. Maltby J. Baliousis M. Joseph S. (2008). The authentic personality: A theoretical and empirical conceptualization and the development of the authenticity scale. Journal of Counseling Psychology, 55(3), 385–399. 10.1037/0022-0167.55.3.385

