# Supplementary material for: Subjective Identity Concealability and the Consequences of Fearing Identity-Based Judgment
Source: Pers Soc Psychol Bull. 2021 Apr 23;48(3):445–62. doi: 10.1177/01461672211010038 (PMC8855390; doi:10.1177/01461672211010038)
Supplement: sj-docx-1-psp-10.1177_01461672211010038 – Supplemental material for Subjective Identity Concealability and the Consequences of Fearing Identity-Based Judgment [file sj-docx-1-psp-10.1177_01461672211010038.docx]

Subjective Identity Concealability and the Consequences of Fearing Identity-Based Judgment: Methodology File

**Study 1**

**Procedure Overview**

- Participants consent to take the study
- Participants complete a commitment device adapted from Zhou & Fishbach (2016)
- Participants answer open- and closed-ended questions meant to elicit some of their identities and information about how concealable they view those identities as being, partially following a procedure adapted from Grossack (1960)
- Participants complete an Implicit Association Test (as an educational opportunity for participants only; the IAT does not directly relate to the research question being investigated)
  - Technical details for the IAT are not included, because its data will not be analyzed
- Participants complete a demographic questionnaire
- Participants are debriefed

**Procedure Step-by-Step**

**Commitment Device**

In this study, you will take a questionnaire and an Implicit Association Test (IAT). An IAT is a sorting task where you will be asked to sort pictures and words into different categories.

This session will take approximately 10-15 minutes to complete. Many people may be tempted to visit other web pages while taking the study. **If a lot of people browse other pages or do other things during the study, the study's data won't be usable. However, our research depends on good quality data.** So, please make sure you are willing to sit through the study before starting it.

If you would like to participate, please type this exact sentence into the box below: **"I will complete this study with my full attention."** and press "Submit".

[commit]

*Text response.*

**Open- and Closed-Ended Questions**

**Please write three answers to the question: “Who am I?” in the blanks.**

Answer as if you were giving the answers to yourself, not to somebody else. Try to provide your answers in single words or short phrases, if possible.

[identity1]

*Text response.*

[identity2]

*Text response.*

[identity3]

*Text response.*

**Please answer each of the following questions:**

In general, how easy or hard would it be to hide that you are a {response: identity1) if you wanted to?

[conceal1]

- *Very easy*
- *Moderately easy*
- *Somewhat easy*
- *Neither easy nor hard*
- *Somewhat hard*
- *Moderately hard*
- *Very hard*

In general, how easy or hard would it be to hide that you are a {response: identity2) if you wanted to?

[conceal2]

- *Very easy*
- *Moderately easy*
- *Somewhat easy*
- *Neither easy nor hard*
- *Somewhat hard*
- *Moderately hard*
- *Very hard*

In general, how easy or hard would it be to hide that you are a {response: identity3) if you wanted to?

[conceal3]

- *Very easy*
- *Moderately easy*
- *Somewhat easy*
- *Neither easy nor hard*
- *Somewhat hard*
- *Moderately hard*
- *Very hard*

*The order of the following conceal_easy and conceal_hard questions is counterbalanced, so that participants either see all three easy questions first, followed by all three hard questions, or all three hard questions first, followed by all three easy questions.*

**Please answer the following questions using as few words as possible.**

If you cannot think of an answer, feel free to leave some questions blank.

It’s **easy** to conceal that I am a (response:identity1) because…

[conceal_easy1]

*Text response.*

It’s **easy** to conceal that I am a (response:identity2) because…

[conceal_easy2]

*Text response.*

It’s **easy** to conceal that I am a (response:identity3) because…

[conceal_easy3]

*Text response.*

**Please answer the following questions using as few words as possible.**

If you cannot think of an answer, feel free to leave some questions blank.

It’s **hard** to conceal that I am a (response:identity1) because…

[conceal_hard1]

*Text response.*

It’s **hard** to conceal that I am a (response:identity2) because…

[conceal_hard2]

*Text response.*

It’s **hard** to conceal that I am a (response:identity3) because…

[conceal_hard3]

*Text response.*

**Implicit Association Test**

**Stimuli:**

**Gay People:**
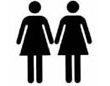
, Gay people, Homosexuals, Lesbians, Gay women

**Straight People:**
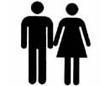
, Straight, Straight People, Heterosexuals

**Good:** Joy, Glorious, Wonderful, Love, Happy, Laughter, Excellent, Great

**Bad:** Terrible, Nasty, Evil, Hurt, Horrible, Failure, Awful, Agony


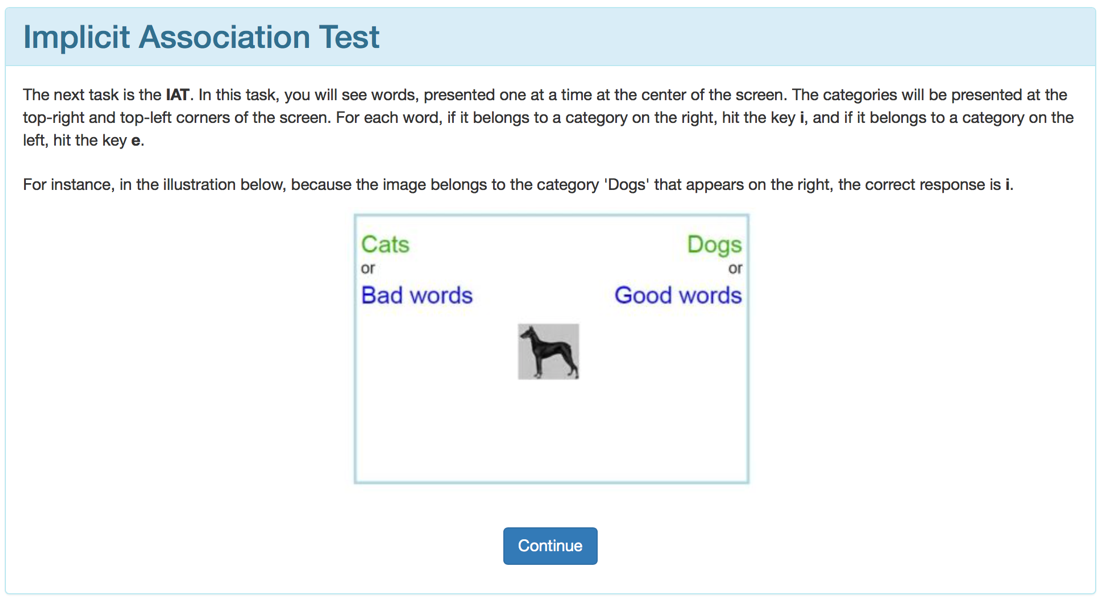


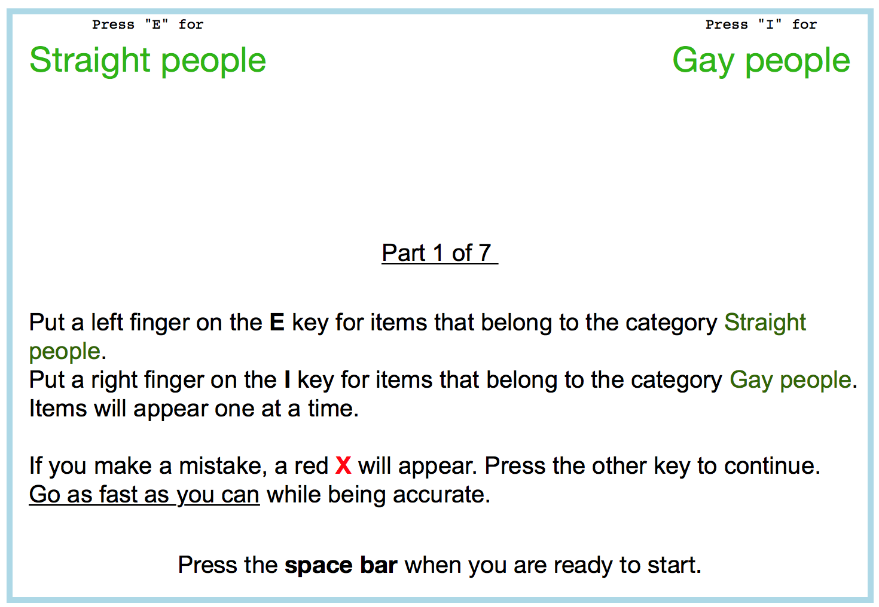


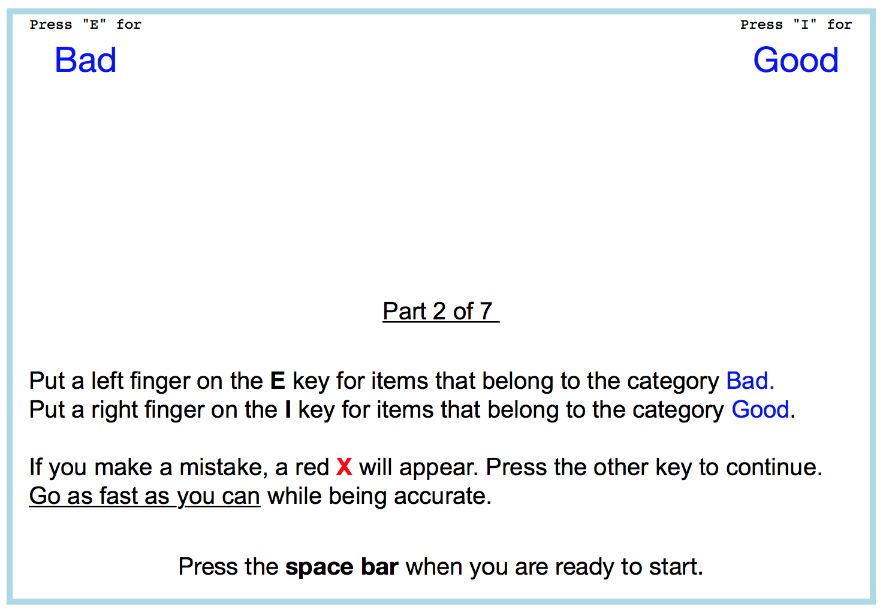


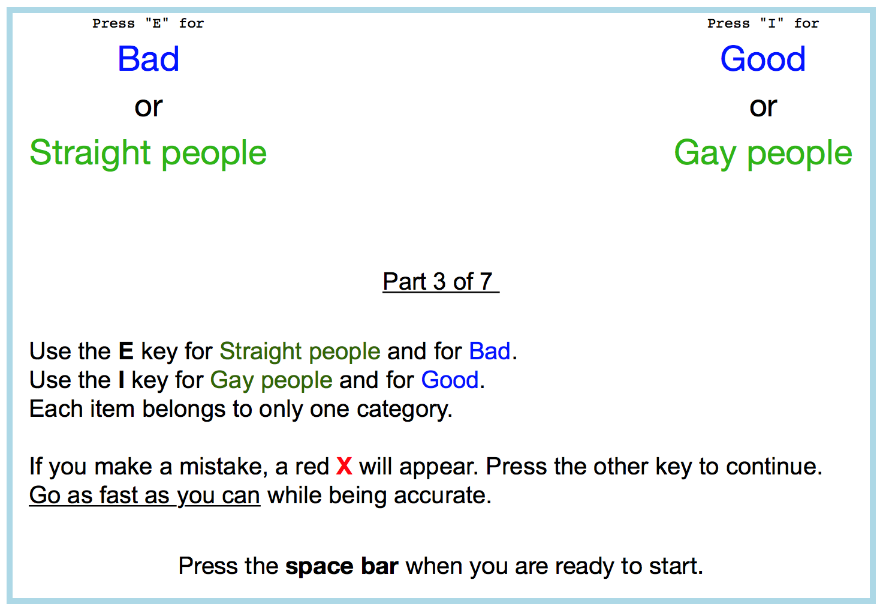


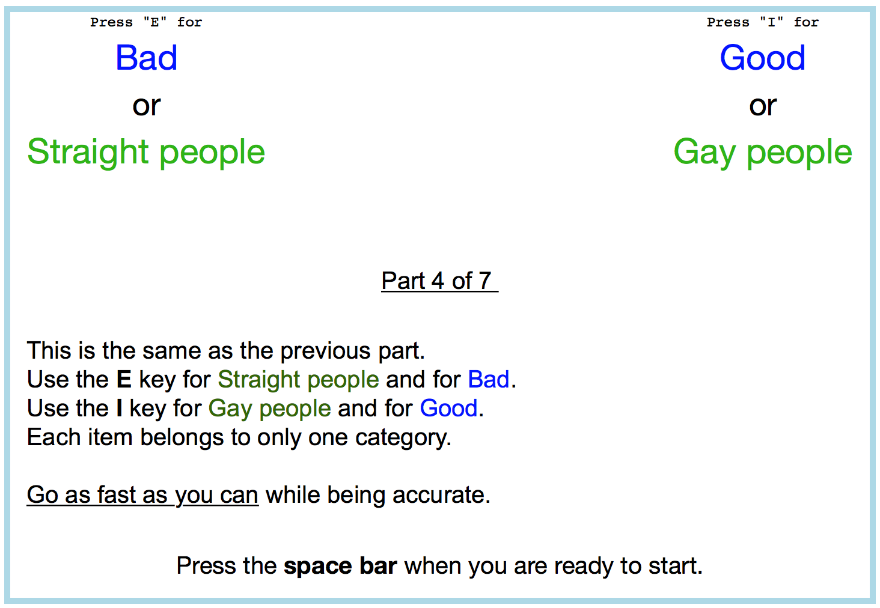


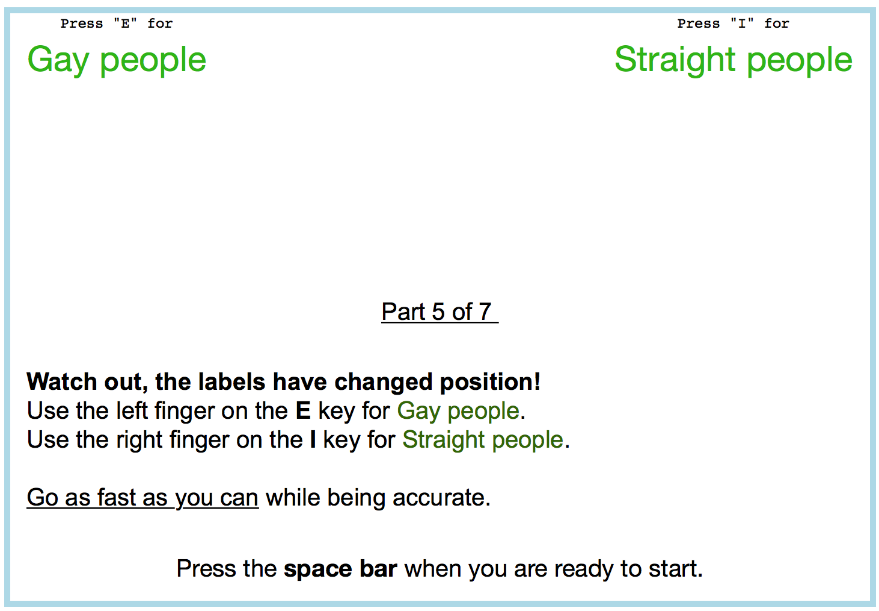


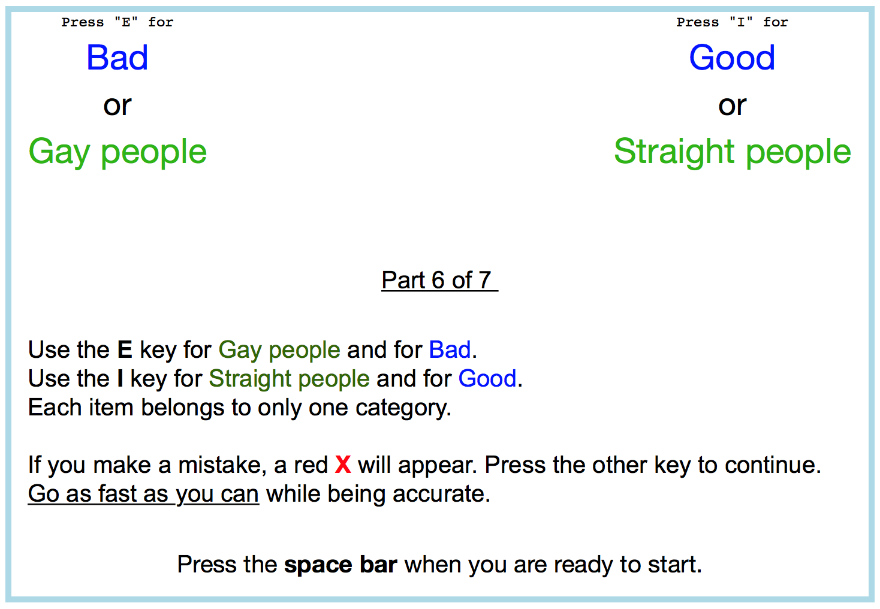


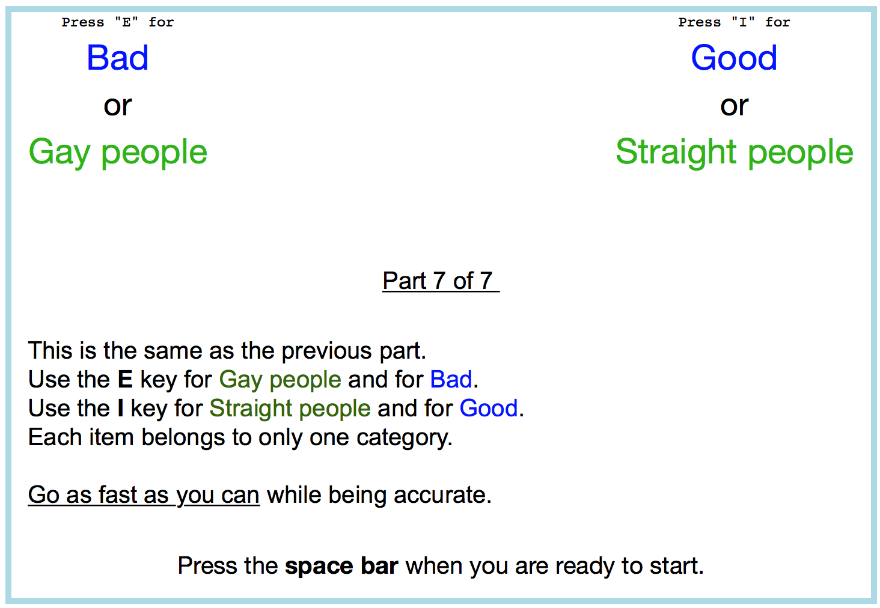


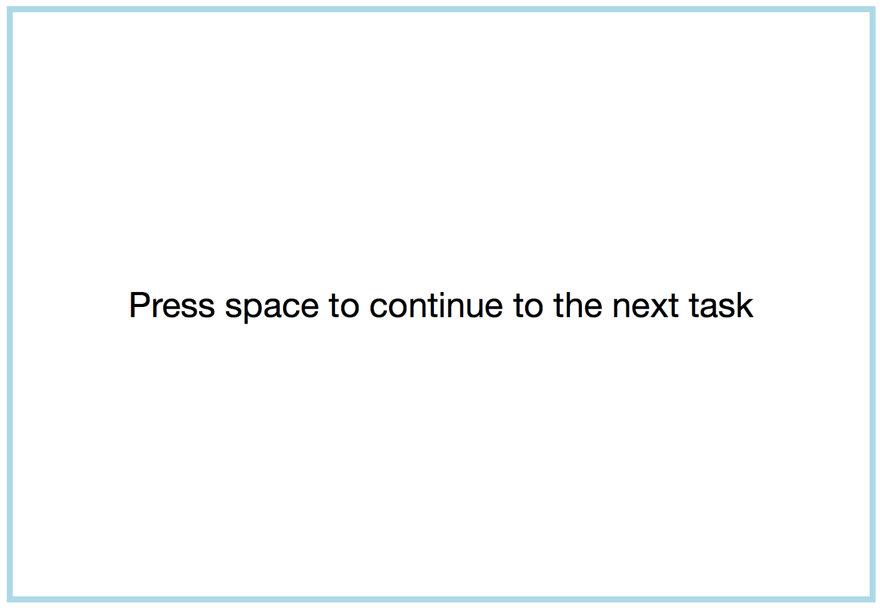


**Demographic Questions**

What is your age?

[age]

*Drop-down menu with response options from 10-100.*

What was your sex, as assigned at birth?

[sex]

- *Male*
- *Female*
- *Other*

Do you consider yourself to be transgender, transsexual, or another member of the gender minority community?

[trans]

- *Yes*
- *No*
- *Unsure*

What is your race?

[race]

- *Asian or Pacific Islander*
- *Black or African American*
- *Hispanic or Latino*
- *Native American*
- *White*
- *Other*

Please indicate your ethnic origin by choosing one of the ten categories listed below. Ethnic origin refers to the ethnic or cultural group(s) to which your recent ancestors belonged. Ethnic origin pertains to ancestral identity or background and should not be confused with citizenship or nationality. If you have multiple ethnic origins, then please select the one with which you most strongly identify. If this is not possible, or if you feel that none of the categories applies to you, then choose “Other”.

[ethnic]

- *European (including British Isles)*
- *East and Southeast Asian (e.g., China, Japan, Korea, Vietnam)*
- *South Asian (e.g., India, Pakistan, Bangladesh, Sri Lanka)*
- *Middle Eastern*
- *African*
- *Latin, Central, and South American*
- *Caribbean*
- *Pacific Islands*
- *Aboriginal*
- *Other*

The terms "mixed-race" and "biracial" refer to people whose mother and father belong to different racial groups (e.g., one parent is Black and the other parent is Asian). With this in mind, are you multiracial, mixed-race or biracial?

[biracial]

- *Yes, I consider myself to be mixed-race or biracial*
- *No, I do not consider myself to be mixed-race or biracial*

What is your sexual orientation?

[orientation]

- *Straight (heterosexual)*
- *Gay (homosexual)*
- *Bisexual or Pansexual*
- *Asexual*
- *Queer or Other*

How urban or rural is the place where you live?

[urban]

- *Very urban*
- *Moderately urban*
- *Somewhat urban*
- *Neither urban nor rural*
- *Somewhat rural*
- *Moderately rural*
- *Very rural*

**Additional Variables to be Extracted from Project Implicit Prescreening:**

Religion:

[religion]

- Buddhist/Confucian/Shinto
- Christian: Catholic or Orthodox
- Christian: Protestant or Other
- Hindu
- Jewish
- Muslim/Islamic
- Not Religious
- Other Religion

Religiosity:

[religiosity]

- *Very Religious*
- *Moderately Religious*
- *Somewhat Religious*
- *Not at all Religious*

Political Identity:

[political]

- *Strongly Liberal*
- *Moderately Liberal*
- *Somewhat Liberal*
- *Neutral (Moderate)*
- *Somewhat Conservative*
- *Moderately Conservative*
- *Strongly Conservative*

**Study 2**

**Study Overview:**

- Participants consent to complete the study
- Participants complete the demographic survey
- Participants complete the piping questions
  - Their response to this question will be used to customize subsequent questionnaires so that we can be confident participants are being asked about participants they actually hold and are interested in concealing
- Participants complete the preliminary Subjective Identity Concealability scale (long version for EFA)
- Participants complete exploratory survey measures

**Demographic Survey**

The following questions will ask you about your demographic characteristics. Please answer honestly.

What is your age?

[d_age]

- *Drop-down menu with response options from 18-100*

What was your sex, as assigned at birth?

[d_sex]

- *Male*
- *Female*
- *Other:__________*

Do you consider yourself to be transgender, transsexual, or another member of the gender minority community?

[d_genderminority]

- *Yes*
- *No*
- *Unsure*

Please indicate your ethnic origin by choosing one of the ten categories listed below. Ethnic origin refers to the ethnic or cultural group(s) to which your recent ancestors belonged. Ethnic origin pertains to ancestral identity or background and should not be confused with citizenship or nationality. If you have multiple ethnic origins, then please select the one with which you most strongly identify. If this is not possible, or if you feel that none of the categories applies to you, then choose “Other”.

[d_ethnicity]

- *European (including British Isles)*
- *East and Southeast Asian (e.g., China, Japan, Korea, Vietnam)*
- *South Asian (e.g., India, Pakistan, Bangladesh, Sri Lanka)*
- *Middle Eastern*
- *African*
- *Latin, Central, and South American*
- *Caribbean*
- *Pacific Islander*
- *Aboriginal*
- *Other:__________*

The terms “multiracial”, "mixed-race", and "biracial" refer to people whose mother and father belong to different racial groups (e.g., one parent is Black and the other parent is Asian). With this in mind, are you multiracial, mixed-race or biracial?

[d_biracial]

- *Yes, I consider myself to be multiracial, mixed-race, or biracial*
- *No, I do not consider myself to be multiracial, mixed-race, or biracial*

What is your sexual orientation?

[d_sexuality]

- *Straight (heterosexual)*
- *Gay (homosexual)*
- *Bisexual/Pansexual*
- *Asexual*
- *Queer*
- *Other:__________*

How urban or rural is the place where you live?

[d_urban]

- *Very urban*
- *Moderately urban*
- *Somewhat urban*
- *Neither urban nor rural*
- *Somewhat rural*
- *Moderately rural*
- *Very rural*

What is your religion?

[d_religion]

- *Buddhist*
- *Christian: Catholic or Orthodox*
- *Christian: Protestant or Other*
- *Hindu*
- *Jewish*
- *Muslim*
- *Shintoist*
- *Sikh*
- *Taoist*
- *Atheist/Agnostic/Not religious*
- *Other:__________*

How religious are you?

[d_religiosity]

- *Very Religious*
- *Moderately Religious*
- *Somewhat Religious*
- *Not at all Religious*

How would you describe your political views?

[d_political]

- *Very Liberal*
- *Moderately Liberal*
- *Somewhat Liberal*
- *Moderate (neither Liberal nor Conservative)*
- *Somewhat Conservative*
- *Moderately Conservative*
- *Very Conservative*

What is your nationality?

[d_nationality]

- *Afghan*
- *Albanian*
- *Algerian*
- *American*
- *Andorran*
- *Angolan*
- *Antiguans*
- *Argentinean*
- *Armenian*
- *Australian*
- *Austrian*
- *Azerbaijani*
- *Bahamian*
- *Bahraini*
- *Bangladeshi*
- *Barbadian*
- *Barbudans*
- *Batswana*
- *Belarusian*
- *Belgian*
- *Belizean*
- *Beninese*
- *Bhutanese*
- *Bolivian*
- *Bosnian*
- *Brazilian*
- *British*
- *Bruneian*
- *Bulgarian*
- *Burkinabe*
- *Burmese*
- *Burundian*
- *Cambodian*
- *Cameroonian*
- *Canadian*
- *Cape Verdean*
- *Central African*
- *Chadian*
- *Chilean*
- *Chinese*
- *Colombian*
- *Comoran*
- *Congolese*
- *Congolese*
- *Costa Rican*
- *Croatian*
- *Cuban*
- *Cypriot*
- *Czech*
- *Danish*
- *Djibouti*
- *Dominican*
- *Dominican*
- *Dutch*
- *Dutchman*
- *Dutchwoman*
- *East Timorese*
- *Ecuadorean*
- *Egyptian*
- *Emirian*
- *Equatorial Guinean*
- *Eritrean*
- *Estonian*
- *Ethiopian*
- *Fijian*
- *Filipino*
- *Finnish*
- *French*
- *Gabonese*
- *Gambian*
- *Georgian*
- *German*
- *Ghanaian*
- *Greek*
- *Grenadian*
- *Guatemalan*
- *Guinea-Bissauan*
- *Guinean*
- *Guyanese*
- *Haitian*
- *Herzegovinian*
- *Honduran*
- *Hungarian*
- *I-Kiribati*
- *Icelander*
- *Indian*
- *Indonesian*
- *Iranian*
- *Iraqi*
- *Irish*
- *Irish*
- *Israeli*
- *Italian*
- *Ivorian*
- *Jamaican*
- *Japanese*
- *Jordanian*
- *Kazakhstani*
- *Kenyan*
- *Kittian and Nevisian*
- *Kuwaiti*
- *Kyrgyz*
- *Laotian*
- *Latvian*
- *Lebanese*
- *Liberian*
- *Libyan*
- *Liechtensteiner*
- *Lithuanian*
- *Luxembourger*
- *Macedonian*
- *Malagasy*
- *Malawian*
- *Malaysian*
- *Maldivan*
- *Malian*
- *Maltese*
- *Marshallese*
- *Mauritanian*
- *Mauritian*
- *Mexican*
- *Micronesian*
- *Moldovan*
- *Monacan*
- *Mongolian*
- *Moroccan*
- *Mosotho*
- *Motswana*
- *Mozambican*
- *Namibian*
- *Nauruan*
- *Nepalese*
- *Netherlander*
- *New Zealander*
- *Ni-Vanuatu*
- *Nicaraguan*
- *Nigerian*
- *Nigerien*
- *North Korean*
- *Northern Irish*
- *Norwegian*
- *Omani*
- *Pakistani*
- *Palauan*
- *Panamanian*
- *Papua New Guinean*
- *Paraguayan*
- *Peruvian*
- *Polish*
- *Portuguese*
- *Qatari*
- *Romanian*
- *Russian*
- *Rwandan*
- *Saint Lucian*
- *Salvadoran*
- *Samoan*
- *San Marinese*
- *Sao Tomean*
- *Saudi*
- *Scottish*
- *Senegalese*
- *Serbian*
- *Seychellois*
- *Sierra Leonean*
- *Singaporean*
- *Slovakian*
- *Slovenian*
- *Solomon Islander*
- *Somali*
- *South African*
- *South Korean*
- *Spanish*
- *Sri Lankan*
- *Sudanese*
- *Surinamer*
- *Swazi*
- *Swedish*
- *Swiss*
- *Syrian*
- *Taiwanese*
- *Tajik*
- *Tanzanian*
- *Thai*
- *Togolese*
- *Tongan*
- *Trinidadian or Tobagonian*
- *Tunisian*
- *Turkish*
- *Tuvaluan*
- *Ugandan*
- *Ukrainian*
- *Uruguayan*
- *Uzbekistani*
- *Venezuelan*
- *Vietnamese*
- *Welsh*
- *Yemenite*
- *Zambian*
- *Zimbabwean*

In which country do you *currently* live?

[d_residence]

- *Afghanistan*
- *Albania*
- *Algeria*
- *American Samoa*
- *Andorra*
- *Angola*
- *Anguilla*
- *Antigua And Barbuda*
- *Argentina*
- *Armenia*
- *Aruba*
- *Australia*
- *Austria*
- *Azerbaijan*
- *Bahamas*
- *Bahrain*
- *Bangladesh*
- *Barbados*
- *Belarus*
- *Belgium*
- *Belize*
- *Benin*
- *Bermuda*
- *Bhutan*
- *Bolivia*
- *Bosnia And Herzegovina*
- *Botswana*
- *Bouvet Island*
- *Brazil*
- *British Indian Ocean Territory*
- *Brunei Darussalam*
- *Bulgaria*
- *Burkina Faso*
- *Burundi*
- *Cambodia*
- *Cameroon*
- *Canada*
- *Cape Verde*
- *Cayman Islands*
- *Central African Republic*
- *Chad*
- *Chile*
- *China*
- *Christmas Island*
- *Cocos (keeling) Islands*
- *Colombia*
- *Comoros*
- *Congo*
- *The Democratic Republic Of The Congo*
- *Cook Islands*
- *Costa Rica*
- *Cote D'Ivoire*
- *Croatia*
- *Cuba*
- *Cyprus*
- *Czech Republic*
- *Denmark*
- *Djibouti*
- *Dominica*
- *Dominican Republic*
- *East Timor*
- *Ecuador*
- *Egypt*
- *El Salvador*
- *Equatorial Guinea*
- *Eritrea*
- *Estonia*
- *Ethiopia*
- *Falkland Islands (Malvinas)*
- *Faroe Islands*
- *Fiji*
- *Finland*
- *France*
- *French Guiana*
- *French Polynesia*
- *French Southern Territories*
- *Gabon*
- *Gambia*
- *Georgia*
- *Germany*
- *Ghana*
- *Gibraltar*
- *Greece*
- *Greenland*
- *Grenada*
- *Guadeloupe*
- *Guam*
- *Guatemala*
- *Guinea*
- *Guinea-Bissau*
- *Guyana*
- *Haiti*
- *Heard Island And McDonald Islands*
- *Holy See (Vatican City State)*
- *Honduras*
- *Hong Kong*
- *Hungary*
- *Iceland*
- *India*
- *Indonesia*
- *Iran, Islamic Republic Of*
- *Iraq*
- *Ireland*
- *Israel*
- *Italy*
- *Jamaica*
- *Japan*
- *Jordan*
- *Kazakhstan*
- *Kenya*
- *Kiribati*
- *Korea, Democratic People's Republic Of*
- *Korea, Republic Of*
- *Kosovo*
- *Kuwait*
- *Kyrgyzstan*
- *Lao People's Democratic Republic*
- *Latvia*
- *Lebanon*
- *Lesotho*
- *Liberia*
- *Libyan Arab Jamahiriya*
- *Liechtenstein*
- *Lithuania*
- *Luxembourg*
- *Macau*
- *Macedonia, The Former Yugoslav Republic Of*
- *Madagascar*
- *Malawi*
- *Malaysia*
- *Maldives*
- *Mali*
- *Malta*
- *Marshall Islands*
- *Martinique*
- *Mauritania*
- *Mauritius*
- *Mayotte*
- *Mexico*
- *Micronesia, Federated States Of*
- *Moldova, Republic Of*
- *Monaco*
- *Mongolia*
- *Montserrat*
- *Montenegro*
- *Morocco*
- *Mozambique*
- *Myanmar*
- *Namibia*
- *Nauru*
- *Nepal*
- *Netherlands*
- *Netherlands Antilles*
- *New Caledonia*
- *New Zealand*
- *Nicaragua*
- *Niger*
- *Nigeria*
- *Niue*
- *Norfolk Island*
- *Northern Mariana Islands*
- *Norway*
- *Oman*
- *Pakistan*
- *Palau*
- *Palestinian Territory*
- *Occupied*
- *Panama*
- *Papua New Guinea*
- *Paraguay*
- *Peru*
- *Philippines*
- *Pitcairn*
- *Poland*
- *Portugal*
- *Puerto Rico*
- *Qatar*
- *Reunion*
- *Romania*
- *Russian Federation*
- *Rwanda*
- *Saint Helena*
- *Saint Kitts And Nevis*
- *Saint Lucia*
- *Saint Pierre And Miquelon*
- *Saint Vincent And The Grenadines*
- *Samoa*
- *San Marino*
- *Sao Tome And Principe*
- *Saudi Arabia*
- *Senegal*
- *Serbia*
- *Seychelles*
- *Sierra Leone*
- *Singapore*
- *Slovakia*
- *Slovenia*
- *Solomon Islands*
- *Somalia*
- *South Africa*
- *South Georgia And The South Sandwich Islands*
- *Spain*
- *Sri Lanka*
- *Sudan*
- *Suriname*
- *Svalbard And Jan Mayen*
- *Swaziland*
- *Sweden*
- *Switzerland*
- *Syrian Arab Republic*
- *Taiwan, Province Of China*
- *Tajikistan*
- *Tanzania, United Republic Of*
- *Thailand*
- *Togo*
- *Tokelau*
- *Tonga*
- *Trinidad And Tobago*
- *Tunisia*
- *Turkey*
- *Turkmenistan*
- *Turks And Caicos Islands*
- *Tuvalu*
- *Uganda*
- *Ukraine*
- *United Arab Emirates*
- *United Kingdom*
- *United States*
- *United States Minor Outlying Islands*
- *Uruguay*
- *Uzbekistan*
- *Vanuatu*
- *Venezuela*
- *Viet Nam*
- *Virgin Islands, British*
- *Virgin Islands, U.S.*
- *Wallis And Futuna*
- *Western Sahara*
- *Yemen*
- *Zambia*
- *Zimbabwe*
- *Other*

**Questions for Piping**

Sometimes, people wish they could conceal a part of themselves from others. That is, they wish they could control whether others knew a specific piece of information about them. Please read through this list of identities and choose one that you have sometimes felt that you wanted or needed to conceal from others.

[conceal_id]

- *your age*
- *your ethnicity*
- *your gender identity*
- *your job*
- *your nationality*
- *your political ideology*
- *your race*
- *your religion*
- *your sex*
- *your sexual orientation*

What specific label or name would you use to describe [response: conceal_id]?

Please do not include words or phrases other than the identity name in this box. For example, if you selected "Race" and you are White, please only write "White" in the box below, not "I am White", "White person", or any other phrase. Thank you.

[pipe]

*Text response*

**Survey Measures Introduction**

*In all the following instructions and questions, the “X”s are replaced by participants’ responses to “pipe”. So, for a White participant who chose “Race” as their response to conceal_id, future questions would ask them about their identity as a White person.)*

Thank you! Next, you will be presented with a questionnaire about what it’s like to be X.

**Novel Measures of Subjective Identity Concealability**

**Subjective Identity Concealability Scale Items**

*Each of the following questions are answered on a 5-point scale anchored by the response options indicated in italics beneath the question.*

*In this study, these will be presented in random order.*

Please take a moment to consider your identity as a X person. Think about how the fact that you are X affects you. Think about what it is like to be X. Then, answer the following questions:

1. How typical are you of an average X person? *(Reverse scored)*
   1. *Extremely—Not at all*

[sic01]

1. How surprised would most people be to learn that you are X?
   1. *Extremely—Not at all*

[sic02]

1. How good an example of X people are you? *(Reverse scored)*
   1. *Extremely—Not at all*

[sic03]

1. How important a part of who you are is the fact that you are X? *(Reverse scored)*
   1. *Extremely—Not at all*

[sic04]

1. How affected are you by the fact that you are X? *(Reverse scored)*
   1. *Extremely—Not at all*

[sic05]

1. How much does being X define who you are? *(Reverse scored)*
   1. *Extremely—Not at all*

[sic06]

1. How much do you like being X? *(Reverse scored)*
   1. *Extremely—Not at all*

[sic07]

1. Most of the time, how free do you feel to express the fact that you are X? *(Reverse scored)*
   1. *Extremely—Not at all*

[sic08]

1. How often do you do things that make it obvious that you are X to those around you? *(Reverse scored)*
   1. *Extremely—Not at all*

[sic09]

1. How accepting are others of the fact that you are X? *(Reverse scored)*
   1. *Extremely—Not at all*

[sic10]

1. How much does the fact that you are X change from day to day?
   1. *Extremely—Not at all*

[sic11]

1. How well do people tend to guess that you are X even if you don’t tell them? *(Reverse scored)*
   1. *Extremely—Not at all*

[sic12]

1. How often do people ask you if you are X? *(Reverse scored)*
   1. *Extremely—Not at all*

[sic13]

1. How “out” do you consider yourself to be (as in, do people in your life know that you are X)? *(Reverse scored)*
   1. *Extremely—Not at all*

[sic14]

1. How much would people believe you if you said you were not X?
   1. *Extremely—Not at all*

[sic15]

1. How willing are you to alter things about yourself to prevent others from knowing that you are X?
   1. *Extremely—Not at all*

[sic16]

1. How visible is the fact that you are X? *(Reverse scored)*
   1. *Extremely—Not at all*

[sic17]

1. In general, how knowledgeable are people about what it means to be X? *(Reverse scored)*
   1. *Extremely—Not at all*

[sic18]

1. How frequently do people mix up X people with a different type of person?
   1. *Extremely—Not at all*

[sic19]

1. How experienced are you at trying to hide the fact that you are X?
   1. *Extremely—Not at all*

[sic20]

1. How good are you at blending in, so that the fact that you are X doesn't stand out?
   1. *Extremely—Not at all*

[sic21]

1. How able do you feel to act in a way that is the opposite of what people expect from people who are X?
   1. *Extremely—Not at all*

[sic22]

1. How easy is it for you to conceal that you are X?
   1. *Extremely—Not at al*

[sic23]

1. How attentive are people to cues, signs, or signals that you are X? *(Reverse scored)*
   1. *Extremely—Not at all*

[sic24]

1. How frequently do people notice that you are X? *(Reverse scored)*
   1. *Extremely—Not at all*

[sic25]

1. How able do you feel to avoid “letting it slip” that you are X?
   1. *Extremely—Not at all*

[sic26]

1. How quick are people to figure out that you are X? *(Reverse scored)*
   1. *Extremely—Not at all*

[sic27]

1. How much does the fact that you are X make you stand out? *(Reverse scored)*
   1. *Extremely—Not at all*

[sic28]

1. If you wanted to, how able would you be to stop being X?
   1. *Extremely—Not at all*

[sic29]

1. For this question, choose slightly
   1. *Extremely—Not at all*

[attn1]

**Exploratory Measures to inform future study design**

**Threat of Being Stereotyped** (Adapted from Cohen & Garcia, 2005)

*The following question is posed using a 7-point scale anchored by response options Strongly Agree and Strongly Disagree.*

1. I worry that people will draw conclusions about me, based on what they think about X people.

[tbs]

**Personal Experience of Prejudice**

*The following question is posed using a 7-point scale anchored by response options Strongly Agree and Strongly Disagree.*

1. I experience prejudice because I am X.

[expprej]

**Feeling Thermometer (Explicit Attitude Measure)**

1. Please rate how warm or cold you feel toward the following group: X people.
   1. *Very warm*
   2. *Moderately warm*
   3. *Slightly warm*
   4. *Neutral*
   5. *Slightly cold*
   6. *Moderately cold*
   7. *Very cold*

[therm]

**Attention Check 2**

At the start of the survey, you told us which one of your traits you most frequently wished you could conceal. Which trait was it?

[attn_2]

- *your age*
- *your ethnicity*
- *your gender identity*
- *your job*
- *your nationality*
- *your political ideology*
- *your race*
- *your religion*
- *your sex*
- *your sexual orientation*

**Study 3**

**Study Overview:**

- Participants consent to complete the study
- Participants complete the demographic survey
- Participants complete the piping questions
  - Their response to this question will be used to customize subsequent questionnaires so that we can be confident participants are being asked about participants they actually hold and are interested in concealing
- Participants complete the preliminary Subjective Identity Concealability scale items
- Participants complete theoretically-predicted outcome measures
- Participants complete a Race IAT *(included only to satisfy Project Implicit pool requirements, IAT data will not be analyzed)*
- Participants are debriefed

**Demographic Survey**

The following questions will ask you about your demographic characteristics. Please answer honestly.

What is your age?

[d_age]

- *Drop-down menu with response options from 18-100*

What was your sex, as assigned at birth?

[d_sex]

- *Male*
- *Female*
- *Other*

Do you consider yourself to be transgender, transsexual, or another member of the gender minority community?

[d_genderminority]

- *Yes*
- *No*
- *Unsure*

Please indicate your ethnic origin by choosing one of the ten categories listed below. Ethnic origin refers to the ethnic or cultural group(s) to which your recent ancestors belonged. Ethnic origin pertains to ancestral identity or background and should not be confused with citizenship or nationality. If you have multiple ethnic origins, then please select the one with which you most strongly identify. If this is not possible, or if you feel that none of the categories applies to you, then choose “Other”.

[d_ethnicity]

- *European (including British Isles)*
- *East and Southeast Asian (e.g., China, Japan, Korea, Vietnam)*
- *South Asian (e.g., India, Pakistan, Bangladesh, Sri Lanka)*
- *Middle Eastern*
- *African*
- *Latin, Central, and South American*
- *Caribbean*
- *Pacific Islander*
- *Aboriginal*
- *Other*

The terms “multiracial”, "mixed-race", and "biracial" refer to people whose mother and father belong to different racial groups (e.g., one parent is Black and the other parent is Asian). With this in mind, are you multiracial, mixed-race or biracial?

[d_biracial]

- *Yes, I consider myself to be multiracial, mixed-race, or biracial*
- *No, I do not consider myself to be multiracial, mixed-race, or biracial*

What is your sexual orientation?

[d_sexuality]

- *Straight (heterosexual)*
- *Gay (homosexual)*
- *Bisexual/Pansexual*
- *Asexual*
- *Queer*
- *Other*

How urban or rural is the place where you live?

[d_urban]

- *Very urban*
- *Moderately urban*
- *Somewhat urban*
- *Neither urban nor rural*
- *Somewhat rural*
- *Moderately rural*
- *Very rural*

**Questions for Piping:**

Sometimes, people wish they could conceal a part of themselves from others. That is, they wish they could control whether others knew a specific piece of information about them. Please read through this list of identities and choose one that you have sometimes felt that you wanted or needed to conceal from others.

[conceal_id]

- *your age*
- *your job*
- *your political ideology*
- *your religion*
- *your sexual orientation*

What specific label or name would you use to describe [response: conceal_id]?

Please do not include words or phrases other than the identity name in this box. For example, if you selected "Race" and you are White, please only write "White" in the box below, not "I am White", "White person", or any other phrase. Thank you.

[pipe]

*Text response*

**Survey Measures**

*In all the following instructions and questions, the “X”s are replaced by participants’ responses to “pipe”. So, for a White participant who chose “Race” as their response to conceal_id, future questions would ask them about their identity as a White person.)*

*The following questionnaires are presented in random order. Items within each questionnaire are presented in the order in which they are written in this document unless otherwise indicated.*

**Subjective Identity Concealability Scale Items**

*Each of the following questions are answered on a 5-point scale anchored by the response options indicated in italics beneath the question.*

*In this study, these will be presented in random order.*

Please take a moment to consider your identity as a X person. Think about how the fact that you are X affects you. Think about what it is like to be X. Then, answer the following questions:

1. How much does being X define who you are? *(Reverse scored)*
   1. *Extremely—Not at all*

[sic01]

1. How often do you do things that make it obvious that you are X to those around you? *(Reverse scored)*
   1. *Extremely—Not at all*

[sic02]

1. How well do people tend to guess that you are X even if you don’t tell them? *(Reverse scored)*
   1. *Extremely—Not at all*

[sic03]

1. How “out” do you consider yourself to be (as in, do people in your life know that you are X)? *(Reverse scored)*
   1. *Extremely—Not at all*

[sic04]

1. How visible is the fact that you are X? *(Reverse scored)*
   1. *Extremely—Not at all*

[sic05]

1. How easy is it for you to conceal that you are X?
   1. *Extremely—Not at al*

[sic06]

1. How attentive are people to cues, signs, or signals that you are X? *(Reverse scored)*
   1. *Extremely—Not at all*

[sic07]

1. How frequently do people notice that you are X? *(Reverse scored)*
   1. *Extremely—Not at all*

[sic08]

1. How quick are people to figure out that you are X? *(Reverse scored)*
   1. *Extremely—Not at all*

[sic09]

1. How much does the fact that you are X make you stand out? *(Reverse scored)*
   1. *Extremely—Not at all*

[sic10]

1. For the question, choose slightly.
   1. *Extremely—Not at all*

[attn1]

**Exploratory Measures to inform future study design**

**Threat of Being Stereotyped** (Adapted from Cohen & Garcia, 2005)

*The following question is posed using a 7-point scale anchored by response options Strongly Agree and Strongly Disagree.*

1. I worry that people will draw conclusions about me, based on what they think about X people.

[tbs]

**Personal Experience of Prejudice**

*The following question is posed using a 7-point scale anchored by response options Strongly Agree and Strongly Disagree.*

1. I experience prejudice because I am X.

[pep]

**Feeling Thermometer (Explicit Attitude Measure)**

1. Please rate how warm or cold you feel toward the following group: X people.
   1. *Very warm*
   2. *Moderately warm*
   3. *Slightly warm*
   4. *Neutral*
   5. *Slightly cold*
   6. *Moderately cold*
   7. *Very cold*

[therm]

**Situational Avoidance**

*The following question is posed using a 7-point scale anchored by response options Strongly Agree and Strongly Disagree.*

1. Because I am X, I sometimes avoid doing things I would otherwise like to do.

[sa1]

1. There are things in my life that I would be more comfortable doing if I were not X.

[sa2]

1. There are things in my life that I would spend more time doing if I were not X.

[sa3]

**Intergroup Anxiety**

*All the questions in this questionnaire have the same prompt (below). Only the response items change, as indicated, for each item.*

Prompt: If you were interacting with a group of people (e.g., talking with them, working on a project with them, etc.) and you were the only X person in the group, how would you feel compared to occasions when you are interacting with other people who are X?

- 1. *Extremely awkward*
  2. *Quite awkward*
  3. *Moderately awkward*
  4. *Slightly awkward*
  5. *Not awkward at all*

[ia01]

- 1. *Extremely self-conscious*
  2. *Quite self-conscious*
  3. *Moderately self-conscious*
  4. *Slightly self-conscious*
  5. *Not self-conscious at all*

[ia02]

1. 1. *Extremely happy*
   2. *Quite happy*
   3. *Moderately happy*
   4. *Slightly happy*
   5. *Not happy at all*

[ia03]

1. 1. *Extremely accepted*
   2. *Quite accepted*
   3. *Moderately accepted*
   4. *Slightly accepted*
   5. *Not accepted at all*

[ia04]

1. 1. *Extremely confident*
   2. *Quite confident*
   3. *Moderately confident*
   4. *Slightly confident*
   5. *Not confident at all*

[ia05]

1. 1. *Extremely irritated*
   2. *Quite irritated*
   3. *Moderately irritated*
   4. *Slightly irritated*
   5. *Not irritated at all*

[ia06]

1. 1. *Extremely impatient*
   2. *Quite impatient*
   3. *Moderately impatient*
   4. *Slightly impatient*
   5. *Not impatient at all*

[ia07]

1. 1. *Extremely defensive*
   2. *Quite defensive*
   3. *Moderate defensive*
   4. *Slightly defensive*
   5. *Not defensive at all*

[ia08]

1. 1. *Extremely suspicious*
   2. *Quite suspicious*
   3. *Moderately suspicious*
   4. *Slightly suspicious*
   5. *Not suspicious at all*

[ia09]

- 1. *Extremely careful*
  2. Quite careful
  3. Moderately careful
  4. Slightly careful
  5. Not careful at all

[ia10]

**Belonging Uncertainty**

*The following question is posed using a 7-point scale anchored by response options Strongly Agree and Strongly Disagree.*

1. Sometimes I feel that I belong, and sometimes I feel that I don’t belong.

[bu1]

1. When something bad happens, I feel that maybe I don’t belong.

[bu2]

1. When something good happens, I feel that I really belong.

[bu3]

**Attention Check 2**

At the start of the survey, you told us which one of your traits you most frequently wished you could conceal. Which trait was it?

- *Your age*
- *Your job*
- *Your political ideology*
- *Your religion*
- *Your sexual orientation*

[attn_2]

**5-Block Implicit Association Test**

*Stimuli shown below. IAT data will not be analyzed for this project.*

**Black People Stimuli:**
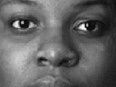

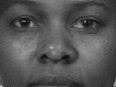

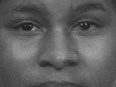

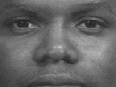

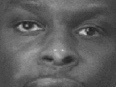

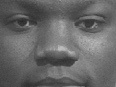


**White People Stimuli:**
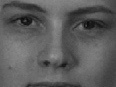

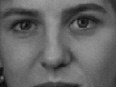

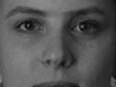

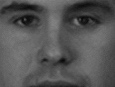

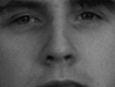

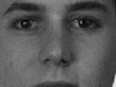


**Good Stimuli:** Pleasing, Cheer, Cheerful, Friendship, Spectacular, Love, Terrific, Attractive

**Bad Stimuli:** Negative, Selfish, Failure, Rotten, Sadness, Detest, Humiliate, Despise

**Questions Provided by PI Prescreen** *(These questions won’t be asked in the main survey because participants will have already answered them in the Project Implicit prescreen.)*

What is your religion?

[d_religion]

- *Buddhist*
- *Christian: Catholic or Orthodox*
- *Christian: Protestant or Other*
- *Hindu*
- *Jewish*
- *Muslim*
- *Shintoist*
- *Sikh*
- *Taoist*
- *Atheist/Agnostic/Not religious*
- *Other:__________*

How religious are you?

[d_religiosity]

- *Very Religious*
- *Moderately Religious*
- *Somewhat Religious*
- *Not at all Religious*

How would you describe your political views?

[d_political]

- *Very Liberal*
- *Moderately Liberal*
- *Somewhat Liberal*
- *Moderate (neither Liberal nor Conservative)*
- *Somewhat Conservative*
- *Moderately Conservative*
- *Very Conservative*

What is your nationality?

[d_nationality]

- *Afghan*
- *Albanian*
- *Algerian*
- *American*
- *Andorran*
- *Angolan*
- *Antiguans*
- *Argentinean*
- *Armenian*
- *Australian*
- *Austrian*
- *Azerbaijani*
- *Bahamian*
- *Bahraini*
- *Bangladeshi*
- *Barbadian*
- *Barbudans*
- *Batswana*
- *Belarusian*
- *Belgian*
- *Belizean*
- *Beninese*
- *Bhutanese*
- *Bolivian*
- *Bosnian*
- *Brazilian*
- *British*
- *Bruneian*
- *Bulgarian*
- *Burkinabe*
- *Burmese*
- *Burundian*
- *Cambodian*
- *Cameroonian*
- *Canadian*
- *Cape Verdean*
- *Central African*
- *Chadian*
- *Chilean*
- *Chinese*
- *Colombian*
- *Comoran*
- *Congolese*
- *Congolese*
- *Costa Rican*
- *Croatian*
- *Cuban*
- *Cypriot*
- *Czech*
- *Danish*
- *Djibouti*
- *Dominican*
- *Dominican*
- *Dutch*
- *Dutchman*
- *Dutchwoman*
- *East Timorese*
- *Ecuadorean*
- *Egyptian*
- *Emirian*
- *Equatorial Guinean*
- *Eritrean*
- *Estonian*
- *Ethiopian*
- *Fijian*
- *Filipino*
- *Finnish*
- *French*
- *Gabonese*
- *Gambian*
- *Georgian*
- *German*
- *Ghanaian*
- *Greek*
- *Grenadian*
- *Guatemalan*
- *Guinea-Bissauan*
- *Guinean*
- *Guyanese*
- *Haitian*
- *Herzegovinian*
- *Honduran*
- *Hungarian*
- *I-Kiribati*
- *Icelander*
- *Indian*
- *Indonesian*
- *Iranian*
- *Iraqi*
- *Irish*
- *Irish*
- *Israeli*
- *Italian*
- *Ivorian*
- *Jamaican*
- *Japanese*
- *Jordanian*
- *Kazakhstani*
- *Kenyan*
- *Kittian and Nevisian*
- *Kuwaiti*
- *Kyrgyz*
- *Laotian*
- *Latvian*
- *Lebanese*
- *Liberian*
- *Libyan*
- *Liechtensteiner*
- *Lithuanian*
- *Luxembourger*
- *Macedonian*
- *Malagasy*
- *Malawian*
- *Malaysian*
- *Maldivan*
- *Malian*
- *Maltese*
- *Marshallese*
- *Mauritanian*
- *Mauritian*
- *Mexican*
- *Micronesian*
- *Moldovan*
- *Monacan*
- *Mongolian*
- *Moroccan*
- *Mosotho*
- *Motswana*
- *Mozambican*
- *Namibian*
- *Nauruan*
- *Nepalese*
- *Netherlander*
- *New Zealander*
- *Ni-Vanuatu*
- *Nicaraguan*
- *Nigerian*
- *Nigerien*
- *North Korean*
- *Northern Irish*
- *Norwegian*
- *Omani*
- *Pakistani*
- *Palauan*
- *Panamanian*
- *Papua New Guinean*
- *Paraguayan*
- *Peruvian*
- *Polish*
- *Portuguese*
- *Qatari*
- *Romanian*
- *Russian*
- *Rwandan*
- *Saint Lucian*
- *Salvadoran*
- *Samoan*
- *San Marinese*
- *Sao Tomean*
- *Saudi*
- *Scottish*
- *Senegalese*
- *Serbian*
- *Seychellois*
- *Sierra Leonean*
- *Singaporean*
- *Slovakian*
- *Slovenian*
- *Solomon Islander*
- *Somali*
- *South African*
- *South Korean*
- *Spanish*
- *Sri Lankan*
- *Sudanese*
- *Surinamer*
- *Swazi*
- *Swedish*
- *Swiss*
- *Syrian*
- *Taiwanese*
- *Tajik*
- *Tanzanian*
- *Thai*
- *Togolese*
- *Tongan*
- *Trinidadian or Tobagonian*
- *Tunisian*
- *Turkish*
- *Tuvaluan*
- *Ugandan*
- *Ukrainian*
- *Uruguayan*
- *Uzbekistani*
- *Venezuelan*
- *Vietnamese*
- *Welsh*
- *Yemenite*
- *Zambian*
- *Zimbabwean*

In which country do you *currently* live?

[d_residence]

- *Afghanistan*
- *Albania*
- *Algeria*
- *American Samoa*
- *Andorra*
- *Angola*
- *Anguilla*
- *Antigua And Barbuda*
- *Argentina*
- *Armenia*
- *Aruba*
- *Australia*
- *Austria*
- *Azerbaijan*
- *Bahamas*
- *Bahrain*
- *Bangladesh*
- *Barbados*
- *Belarus*
- *Belgium*
- *Belize*
- *Benin*
- *Bermuda*
- *Bhutan*
- *Bolivia*
- *Bosnia And Herzegovina*
- *Botswana*
- *Bouvet Island*
- *Brazil*
- *British Indian Ocean Territory*
- *Brunei Darussalam*
- *Bulgaria*
- *Burkina Faso*
- *Burundi*
- *Cambodia*
- *Cameroon*
- *Canada*
- *Cape Verde*
- *Cayman Islands*
- *Central African Republic*
- *Chad*
- *Chile*
- *China*
- *Christmas Island*
- *Cocos (keeling) Islands*
- *Colombia*
- *Comoros*
- *Congo*
- *The Democratic Republic Of The Congo*
- *Cook Islands*
- *Costa Rica*
- *Cote D'Ivoire*
- *Croatia*
- *Cuba*
- *Cyprus*
- *Czech Republic*
- *Denmark*
- *Djibouti*
- *Dominica*
- *Dominican Republic*
- *East Timor*
- *Ecuador*
- *Egypt*
- *El Salvador*
- *Equatorial Guinea*
- *Eritrea*
- *Estonia*
- *Ethiopia*
- *Falkland Islands (Malvinas)*
- *Faroe Islands*
- *Fiji*
- *Finland*
- *France*
- *French Guiana*
- *French Polynesia*
- *French Southern Territories*
- *Gabon*
- *Gambia*
- *Georgia*
- *Germany*
- *Ghana*
- *Gibraltar*
- *Greece*
- *Greenland*
- *Grenada*
- *Guadeloupe*
- *Guam*
- *Guatemala*
- *Guinea*
- *Guinea-Bissau*
- *Guyana*
- *Haiti*
- *Heard Island And McDonald Islands*
- *Holy See (Vatican City State)*
- *Honduras*
- *Hong Kong*
- *Hungary*
- *Iceland*
- *India*
- *Indonesia*
- *Iran, Islamic Republic Of*
- *Iraq*
- *Ireland*
- *Israel*
- *Italy*
- *Jamaica*
- *Japan*
- *Jordan*
- *Kazakhstan*
- *Kenya*
- *Kiribati*
- *Korea, Democratic People's Republic Of*
- *Korea, Republic Of*
- *Kosovo*
- *Kuwait*
- *Kyrgyzstan*
- *Lao People's Democratic Republic*
- *Latvia*
- *Lebanon*
- *Lesotho*
- *Liberia*
- *Libyan Arab Jamahiriya*
- *Liechtenstein*
- *Lithuania*
- *Luxembourg*
- *Macau*
- *Macedonia, The Former Yugoslav Republic Of*
- *Madagascar*
- *Malawi*
- *Malaysia*
- *Maldives*
- *Mali*
- *Malta*
- *Marshall Islands*
- *Martinique*
- *Mauritania*
- *Mauritius*
- *Mayotte*
- *Mexico*
- *Micronesia, Federated States Of*
- *Moldova, Republic Of*
- *Monaco*
- *Mongolia*
- *Montserrat*
- *Montenegro*
- *Morocco*
- *Mozambique*
- *Myanmar*
- *Namibia*
- *Nauru*
- *Nepal*
- *Netherlands*
- *Netherlands Antilles*
- *New Caledonia*
- *New Zealand*
- *Nicaragua*
- *Niger*
- *Nigeria*
- *Niue*
- *Norfolk Island*
- *Northern Mariana Islands*
- *Norway*
- *Oman*
- *Pakistan*
- *Palau*
- *Palestinian Territory*
- *Occupied*
- *Panama*
- *Papua New Guinea*
- *Paraguay*
- *Peru*
- *Philippines*
- *Pitcairn*
- *Poland*
- *Portugal*
- *Puerto Rico*
- *Qatar*
- *Reunion*
- *Romania*
- *Russian Federation*
- *Rwanda*
- *Saint Helena*
- *Saint Kitts And Nevis*
- *Saint Lucia*
- *Saint Pierre And Miquelon*
- *Saint Vincent And The Grenadines*
- *Samoa*
- *San Marino*
- *Sao Tome And Principe*
- *Saudi Arabia*
- *Senegal*
- *Serbia*
- *Seychelles*
- *Sierra Leone*
- *Singapore*
- *Slovakia*
- *Slovenia*
- *Solomon Islands*
- *Somalia*
- *South Africa*
- *South Georgia And The South Sandwich Islands*
- *Spain*
- *Sri Lanka*
- *Sudan*
- *Suriname*
- *Svalbard And Jan Mayen*
- *Swaziland*
- *Sweden*
- *Switzerland*
- *Syrian Arab Republic*
- *Taiwan, Province Of China*
- *Tajikistan*
- *Tanzania, United Republic Of*
- *Thailand*
- *Togo*
- *Tokelau*
- *Tonga*
- *Trinidad And Tobago*
- *Tunisia*
- *Turkey*
- *Turkmenistan*
- *Turks And Caicos Islands*
- *Tuvalu*
- *Uganda*
- *Ukraine*
- *United Arab Emirates*
- *United Kingdom*
- *United States*
- *United States Minor Outlying Islands*
- *Uruguay*
- *Uzbekistan*
- *Vanuatu*
- *Venezuela*
- *Viet Nam*
- *Virgin Islands, British*
- *Virgin Islands, U.S.*
- *Wallis And Futuna*
- *Western Sahara*
- *Yemen*
- *Zambia*
- *Zimbabwe*
- *Other*

**Study 4**

**Study Overview:**

- Participants consent to complete the study
- Participants complete the demographic survey
- Participants complete 2 questionnaires in random order. The order of scales within each questionnaire is randomized.
  - Questionnaire 1: items tailored to an identity the participant reported it being desirable to conceal
  - Questionnaire 2: items that do not require tailoring to identity
- Participants are debriefed

**Demographic Survey**

The following questions will ask you about your demographic characteristics. Please answer honestly.

What is your age?

[d_age]

- *Drop-down menu with response options from 10-100*

What was your sex, as assigned at birth?

[d_sex]

- *Male*
- *Female*
- *Other*

Do you consider yourself to be transgender, transsexual, or another member of the gender minority community?

[d_genderminority]

- *Yes*
- *No*
- *Unsure*

Please indicate your ethnic origin by choosing one of the ten categories listed below. Ethnic origin refers to the ethnic or cultural group(s) to which your recent ancestors belonged. Ethnic origin pertains to ancestral identity or background and should not be confused with citizenship or nationality. If you have multiple ethnic origins, then please select the one with which you most strongly identify. If this is not possible, or if you feel that none of the categories applies to you, then choose “Other”.

[d_ethnicity]

- *European (including British Isles)*
- *East and Southeast Asian (e.g., China, Japan, Korea, Vietnam)*
- *South Asian (e.g., India, Pakistan, Bangladesh, Sri Lanka)*
- *Middle Eastern*
- *African*
- *Latin, Central, and South American*
- *Caribbean*
- *Pacific Islander*
- *Aboriginal*
- *Other*

The terms "mixed-race" and "biracial" refer to people whose mother and father belong to different racial groups (e.g., one parent is Black and the other parent is Asian). With this in mind, are you multiracial, mixed-race or biracial?

[d_biracial]

- *Yes, I consider myself to be mixed-race or biracial*
- *No, I do not consider myself to be mixed-race or biracial*

How urban or rural is the place where you live?

[d_urban]

- *Very urban*
- *Moderately urban*
- *Somewhat urban*
- *Neither urban nor rural*
- *Somewhat rural*
- *Moderately rural*
- *Very rural*

What is your religion?

[d_religion]

- *Buddhist*
- *Christian: Catholic or Orthodox*
- *Christian: Protestant or Other*
- *Hindu*
- *Jewish*
- *Muslim*
- *Shintoist*
- *Sikh*
- *Taoist*
- *Atheist/Agnostic/Not religious*
- *Other*

How religious are you?

[d_religiosity]

- *Very Religious*
- *Moderately Religious*
- *Somewhat Religious*
- *Not at all Religious*

How would you describe your political views?

[d_political]

- *Very Liberal*
- *Moderately Liberal*
- *Somewhat Liberal*
- *Moderate (neither Liberal nor Conservative)*
- *Somewhat Conservative*
- *Moderately Conservative*
- *Very Conservative*

What is your sexual orientation?

[d_sexuality]

- *Straight (heterosexual)*
- *Gay (homosexual)*
- *Bisexual/Pansexual*
- *Asexual*
- *Queer*
- *Other*

What is your nationality?

[d_nationality]

- *Afghan*
- *Albanian*
- *Algerian*
- *American*
- *Andorran*
- *Angolan*
- *Antiguans*
- *Argentinean*
- *Armenian*
- *Australian*
- *Austrian*
- *Azerbaijani*
- *Bahamian*
- *Bahraini*
- *Bangladeshi*
- *Barbadian*
- *Barbudans*
- *Batswana*
- *Belarusian*
- *Belgian*
- *Belizean*
- *Beninese*
- *Bhutanese*
- *Bolivian*
- *Bosnian*
- *Brazilian*
- *British*
- *Bruneian*
- *Bulgarian*
- *Burkinabe*
- *Burmese*
- *Burundian*
- *Cambodian*
- *Cameroonian*
- *Canadian*
- *Cape Verdean*
- *Central African*
- *Chadian*
- *Chilean*
- *Chinese*
- *Colombian*
- *Comoran*
- *Congolese*
- *Congolese*
- *Costa Rican*
- *Croatian*
- *Cuban*
- *Cypriot*
- *Czech*
- *Danish*
- *Djibouti*
- *Dominican*
- *Dominican*
- *Dutch*
- *Dutchman*
- *Dutchwoman*
- *East Timorese*
- *Ecuadorean*
- *Egyptian*
- *Emirian*
- *Equatorial Guinean*
- *Eritrean*
- *Estonian*
- *Ethiopian*
- *Fijian*
- *Filipino*
- *Finnish*
- *French*
- *Gabonese*
- *Gambian*
- *Georgian*
- *German*
- *Ghanaian*
- *Greek*
- *Grenadian*
- *Guatemalan*
- *Guinea-Bissauan*
- *Guinean*
- *Guyanese*
- *Haitian*
- *Herzegovinian*
- *Honduran*
- *Hungarian*
- *I-Kiribati*
- *Icelander*
- *Indian*
- *Indonesian*
- *Iranian*
- *Iraqi*
- *Irish*
- *Irish*
- *Israeli*
- *Italian*
- *Ivorian*
- *Jamaican*
- *Japanese*
- *Jordanian*
- *Kazakhstani*
- *Kenyan*
- *Kittian and Nevisian*
- *Kuwaiti*
- *Kyrgyz*
- *Laotian*
- *Latvian*
- *Lebanese*
- *Liberian*
- *Libyan*
- *Liechtensteiner*
- *Lithuanian*
- *Luxembourger*
- *Macedonian*
- *Malagasy*
- *Malawian*
- *Malaysian*
- *Maldivan*
- *Malian*
- *Maltese*
- *Marshallese*
- *Mauritanian*
- *Mauritian*
- *Mexican*
- *Micronesian*
- *Moldovan*
- *Monacan*
- *Mongolian*
- *Moroccan*
- *Mosotho*
- *Motswana*
- *Mozambican*
- *Namibian*
- *Nauruan*
- *Nepalese*
- *Netherlander*
- *New Zealander*
- *Ni-Vanuatu*
- *Nicaraguan*
- *Nigerian*
- *Nigerien*
- *North Korean*
- *Northern Irish*
- *Norwegian*
- *Omani*
- *Pakistani*
- *Palauan*
- *Panamanian*
- *Papua New Guinean*
- *Paraguayan*
- *Peruvian*
- *Polish*
- *Portuguese*
- *Qatari*
- *Romanian*
- *Russian*
- *Rwandan*
- *Saint Lucian*
- *Salvadoran*
- *Samoan*
- *San Marinese*
- *Sao Tomean*
- *Saudi*
- *Scottish*
- *Senegalese*
- *Serbian*
- *Seychellois*
- *Sierra Leonean*
- *Singaporean*
- *Slovakian*
- *Slovenian*
- *Solomon Islander*
- *Somali*
- *South African*
- *South Korean*
- *Spanish*
- *Sri Lankan*
- *Sudanese*
- *Surinamer*
- *Swazi*
- *Swedish*
- *Swiss*
- *Syrian*
- *Taiwanese*
- *Tajik*
- *Tanzanian*
- *Thai*
- *Togolese*
- *Tongan*
- *Trinidadian or Tobagonian*
- *Tunisian*
- *Turkish*
- *Tuvaluan*
- *Ugandan*
- *Ukrainian*
- *Uruguayan*
- *Uzbekistani*
- *Venezuelan*
- *Vietnamese*
- *Welsh*
- *Yemenite*
- *Zambian*
- *Zimbabwean*
- *Other*

**Questionnaires Customized to Identity**

*In this section, questions are customized to identities participants report wishing they could conceal. Participants are first asked to pick which type of identity they wish they could conceal (e.g., race, ethnicity, etc.). Their response to this question is piped into the next question, which asks them to type their specific identity from that category (e.g., race -> White). Their text response to this question is then piped into future questions. Places where participants’ text response are inserted into a question are indicated with an “X”.*

Thank you! In this section, you will be asked questions about specific identities you hold. Please click “Continue” at the bottom of the page to proceed to the questionnaire.

Sometimes, people wish they could conceal a part of themselves from others. That is, they wish they could control whether others knew a specific piece of information about them. Please read through this list of identities and choose one that you have sometimes felt that you wanted or needed to conceal from others.

[customid_1]

- *Your age*
- *Your ethnicity*
- *Your gender identity*
- *Your job*
- *Your nationality*
- *Your political ideology*
- *Your race*
- *Your religion*
- *Your sex*
- *Your sexual orientation*

What specific label or name would you use to describe [response: customid_1]?

Please do not include words or phrases other than the identity name in this box. For example, if you selected "Race" and you are White, **please only write "White"** in the box below, **not** "I am White", "White person", or any other phrase. Thank you.

[customid1_intro]

*Text response.*

**Subjective Identity Concealability Scale**

*Each of the following questions are answered on a 5-point scale anchored by the response options indicated in italics beneath the question. These will be presented in random order.*

Please take a moment to consider your identity as a X person. Think about how the fact that you are X affects you. Think about what it is like to be X. Then, answer the following questions:

1. How typical are you of an average X person? *(Reverse scored)*
   1. *Extremely typical—Not typical at all*

[custid1_sic01]

1. How surprised would most people be to learn that you are X?
   1. *Extremely surprised—Not surprised at all*

[custid1_sic02]

1. How good an example of X people are you? *(Reverse scored)*
   1. *Extremely good—Not good at all*

[custid1_sic03]

1. How important is being X to your self-image? *(Reverse scored)*
   1. *Extremely important—Not important at all*

[custid1_sic04]

1. How affected are you by the fact that you are X? *(Reverse scored)*
   1. *Extremely affected—Not affected at all*

[custid1_sic05]

1. How much does being X define who you are? *(Reverse scored)*
   1. *Extremely—Not at all*

[custid1_sic06]

1. How much do you like being X? *(Reverse scored)*
   1. *Extremely—Not at all*

[custid1_sic07]

1. Most of the time, how free do you feel to express the fact that you are X? *(Reverse scored)*
   1. *Extremely free—Not free at all*

[custid1_sic08]

1. How often do you do things that make it obvious that you are X to those around you? *(Reverse scored)*
   1. *Extremely often—Not often at all*

[custid1_sic09]

1. How accepting are others of the fact that you are X? *(Reverse scored)*
   1. *Extremely accepting—Not accepting at all*

[custid1_sic10]

1. How much does the fact that you are X change from day to day?
   1. *Extremely—Not at all*

[custid1_sic11]

1. How true is the following statement? *People generally don’t know that I am X unless I tell them.*
   1. *Extremely true—Not true at all*

[custid1_sic12]

1. How often do people ask you if you are X? *(Reverse scored)*
   1. *Extremely often—Not often at all*

[custid1_sic13]

1. How true is the following statement? *Most people in my life already know that I am X. (Reverse scored)*
   1. *Extremely true—Not true at all*

[custid1_sic14]

1. How true is the following statement? *If I said I was not X, people would believe me.*
   1. *Extremely true—Not true at all*

[custid1_sic15]

1. How true is the following statement? *I refuse to change how I act, even if it means others will always know that I am X. (Reverse scored)*
   1. *Extremely true—Not true at all*

[custid1_sic16]

1. How visible is the fact that you are X? *(Reverse scored)*
   1. *Extremely visible—Not visible at all*

[custid1_sic17]

1. In general, how knowledgeable are people about what it means to be X? *(Reverse scored)*
   1. *Extremely knowledgeable—Not knowledgeable at all*

[custid1_sic18]

1. How frequently do people mix up X people with a different type of person?
   1. *Extremely frequently—Not frequently at all*

[custid1_sic19]

1. How experienced are you at trying to hide the fact that you are X?
   1. *Extremely experience—Not experienced at all*

[custid1_sic20]

1. How good are you at blending in, so that the fact that you are X doesn't stand out?
   1. *Extremely good—Not good at all*

[custid1_sic21]

1. How easily can you act in a way that is the opposite of what people expect from people who are X?
   1. *Extremely easily—Not easily at all*

[custid1_sic22]

1. How easy is it for you to conceal that you are X?
   1. *Extremely easy—Not easy at all*

[custid1_sic23]

1. How attentive are people to cues, signs, or signals that you are X? *(Reverse scored)*
   1. *Extremely attentive—Not attentive at all*

[custid1_sic24]

1. How frequently do people notice that you are X? *(Reverse scored)*
   1. *Extremely frequently—Not frequently at all*

[custid1_sic25]

1. Do you feel able to avoid “letting it slip” that you are X?
   1. *Extremely able—Not able at all*

[custid1_sic26]

1. How quick are people to figure out that you are X? *(Reverse scored)*
   1. *Extremely quick—Not quick at all*

[custid1_sic27]

1. How much does the fact that you are X make you stand out? *(Reverse scored)*
   1. *Extremely—Not at all*

[custid1_sic28]

1. If you wanted to, how able would you be to stop being X?
   1. *Extremely able—Not able at all*

[custid1_sic29]

**Feeling Thermometer (Explicit Attitude Measure)**

1. Please rate how warm or cold you feel toward the following group: X people.

[custid1_therm]

- 1. *Very warm*
  2. *Moderately warm*
  3. *Slightly warm*
  4. *Neutral*
  5. *Slightly cold*
  6. *Moderately cold*
  7. *Very cold*

**Identity Centrality** (Adapted from Sellers, Rowley, Chavous, Shelton, & Smith, 1997)

*Each of the following questions is posed using a 7-point scale anchored by response options Strongly Agree and Strongly Disagree.*

1. Overall, being X has very little to do with how I feel about myself. *(Reverse scored)*

[custid1_ic1]

1. In general, being X is an important part of my self-image.

[custid1_ic2]

1. Being X is unimportant to my sense of what kind of person I am. *(Reverse scored)*

[custid1_ic3]

1. I have a strong sense of belonging to X people.

[custid1_ic4]

1. I have a strong attachment to other X people.

[custid1_ic5]

1. Being X is an important reflection of who I am.

[custid1_ic6]

1. Being X is not a major factor in my social relationships. *(Reverse scored)*

[custid1_ic7]

**Group Identification** (Adapted from Verkuyten & Yildiz, 2007)

*Each of the following questions is posed using a 7-point scale anchored by response options Strongly Agree and Strongly Disagree.*

1. I identify with X people.

[custid1_gid1]

1. I feel myself to be X.

[custid1_gid2]

1. I feel connected to X people.

[custid1_gid3]

**Concealment Motivation** (Adapted from Mohr & Kendra, 2011)

*Each of the following questions is posed using a 7-point scale anchored by response options Strongly Agree and Strongly Disagree.*

1. I prefer to keep the fact that I am X rather private.

[custid1_cm1]

1. I keep careful control over who knows about the fact that I am X.

[custid1_cm2]

1. The fact that I am X is a very personal and private matter.

[custid1_cm3]

**Prototypicality** (Adapted from Van Knippenberg & van Knippenberg, 2005)

*Each of the following questions is posed using a 7-point scale anchored by response options Strongly Agree and Strongly Disagree.*

1. I am a good example of X people.

[custid1_proto1]

1. I have a lot in common with other X people.

[custid1_proto2]

1. I represent what is characteristic about X people.

[custid1_proto3]

1. I am representative of X people.

[custid1_proto4]

1. I resemble other X people.

[custid1_proto5]

**Concealment Behavior**

*Each of the following questions is posed using a 5-point scale anchored by response options Extremely frequently and Not frequently at all.*

1. How frequently do you choose to try to conceal the fact that you are X?

[custid1_cb1]

1. When asked if you are X, how frequently do you lie and say that you are not X?

[custid1_cb2]

1. How frequently do you choose to act in a way you hope will lead others to believe that you are not X?

[custid1_cb3]

**Collective Self-Esteem Scale** (Adapted from Luhtanen & Crocker, 1992)

*Each of the following questions is posed using a 7-point scale anchored by response options Strongly disagree and Strongly agree.*

1. I am a worthy member of the group “X people”.

[custid1_cse01]

1. I often regret that I belong to the group “X people”. *(Reverse scored)*

[custid1_cse02]

1. Overall, the group “X people” is considered good by others.

[custid1_cse03]

1. Overall, my membership in the group “X people” has very little to do with how I feel about myself. *(Reverse scored)*

[custid1_cse04]

1. I feel I don’t have much to offer to the group “X people”. *(Reverse scored)*

[custid1_cse05]

1. In general, I’m glad to be a member of the group “X people”.

[custid1_cse06]

1. Most people consider the group “X people”, on the average, to be more ineffective than other social groups. *(Reverse scored)*

[custid1_cse07]

1. The group “X people” is an important reflection of who I am.

[custid1_cse08]

1. I am a cooperative participant in the group “X people”.

[custid1_cse09]

1. Overall, I often feel that the group “X people” is not worthwhile. *(Reverse scored)*

[custid1_cse10]

1. In general, others respect the group “X people”.

[custid1_cse11]

1. The group “X people” is unimportant to my sense of what kind of person I am. *(Reverse scored)*

[custid1_cse12]

1. I often feel I’m a useless member of the group “X people”. *(Reverse scored)*

[custid1_cse13]

1. I feel good about the group “X people”.

[custid1_cse14]

1. In general, others think that the group “X people” is unworthy. *(Reverse scored)*

[custid1_cse15]

1. In general, belonging to the group “X people” is an important part of my self-image.

[custid1_cse16]

**Stigma Consciousness Questionnaire** (Adapted from Pinel, 1999)

*Each of the following questions is posed using a 7-point scale anchored by response options Strongly Agree and Strongly Disagree.*

1. Stereotypes about X people have not affected me personally. *(Reverse scored)*

[custid1_sc01]

1. I never worry that my behaviors will be viewed as stereotypical of X people. *(Reverse scored)*

[custid1_sc02]

1. When interacting with people who are not X who know that I am a X person, I feel like they interpret all my behaviors in terms of the fact that I a X person.

[custid1_sc03]

1. Most people who are not X do not judge X people on the basis of them being X. *(Reverse scored)*

[custid1_sc04]

1. My being X does not influence how people who are not X act with me. *(Reverse scored)*

[custid1_sc05]

1. I almost never think about the fact that I am X when I interact with people who are not X. *(Reverse scored)*

[custid1_sc06]

1. My being X does not influence how people act with me. *(Reverse scored)*

[custid1_sc07]

1. Most people who are not X have a lot more prejudice against X people than they actually express.

[custid1_sc08]

1. I often think that people who are not X are unfairly accused of being prejudiced against X people. *(Reverse scored)*

[custid1_sc09]

1. Most people who are not X have a problem viewing X people as equals.

[custid1_sc10]

**Threat of Being Stereotyped** (Adapted from Cohen & Garcia, 2005)

*The following question is posed using a 7-point scale anchored by response options Strongly Agree and Strongly Disagree.*

1. I worry that people will draw conclusions about me, based on what they think about X people.

[custid1_tbs]

**Situational Avoidance**

*Each of the following questions is posed using a 7-point scale anchored by response options Strongly Agree and Strongly Disagree.*

1. Because I am X, I sometimes avoid doing things I would otherwise like to do.

[custid1_sa1]

1. There are things in my life that I would be more comfortable doing if I were not X.

[custid1_sa2]

1. There are things in my life that I would spend more time doing if I were not X.

[custid1_sa3]

**Intergroup Anxiety** (Adapted from Stephan & Stephan, 1985)

*Each of the following questions employs the same question prompt (the first bullet) and are answered on a 5-point scale anchored by the response options indicated in italics beneath the question.*

1. If you were interacting with a group of people (e.g., talking with them, working on a project with them, etc.) and you were the only X person in the group, how would you feel compared to occasions when you are interacting with other people who *are* X?
   1. *Extremely awkward—Not awkward at all*

[custid1_ia01]

- 1. *Extremely self-conscious—Not self-conscious at all*

[custid1_ia02]

- 1. *Extremely happy—Not happy at all (reverse scored)*

[custid1_ia03]

- 1. *Extremely accepted—Not accepted at all (reverse scored)*

[custid1_ia04]

- 1. *Extremely confident—Not confident at all (reverse scored)*

[custid1_ia05]

- 1. *Extremely irritated—Not irritated at all*

[custid1_ia06]

- 1. *Extremely impatient—Not impatient at all*

[custid1_ia07]

- 1. *Extremely defensive—Not defensive at all*

[custid1_ia08]

- 1. *Extremely suspicious—Not suspicious at all*

[custid1_ia09]

- 1. *Extremely careful—Not careful at all*

[custid1_ia10]

**Personal Experience of Prejudice**

*The following question is answered on a 5-point scale anchored by response options Very frequently and Not frequently at all.*

1. I experience prejudice because I am X.

[custid1_pep]

**In-Group Identification** (From Leach, van Zomeren, Zebel, Vilek, Ouwekerk & Spears, 2008)

*Each of the following questions is posed using a 7-point scale anchored by response options Strongly Agree and Strongly Disagree.*

1. I feel a bond with X people.

[custid1_igi01]

1. I feel solidarity with X people.

[custid1_igi02]

1. I feel committed to X people.

[custid1_igi03]

1. I am glad to be X.

[custid1_igi04]

1. I think that X people have a lot to be proud of.

[custid1_igi05]

1. It is pleasant to be X.

[custid1_igi06]

1. Being X gives me a good feeling.

[custid1_igi07]

1. I often think about the fact that I am X.

[custid1_igi08]

1. The fact that I am X is an important part of my identity.

[custid1_igi09]

1. Being X is an important part of how I see myself.

[custid1_igi10]

1. I have a lot in common with the average X person.

[custid1_igi11]

1. I am similar to the average X person.

[custid1_igi12]

1. X people have a lot in common with each other.

[custid1_igi13]

1. X people are very similar to each other.

[custid1_igi14]

**Questionnaire with Items that do not Require Tailoring to Identity**

Next, you will be asked some general questions about yourself. Please click “Continue” at the bottom of the page to proceed to the first questionnaire.

**General Self-Efficacy** (Adapted from Sherer, Maddux, Mercandante, Prentice-Dunn, Jacobs, & Rogers, 1982)

*Each of the following questions is posed using a 7-point scale anchored by response options Strongly Agree and Strongly Disagree.*

1. When I make plans, I am certain I can make them work.

[all_gse01]

1. One of my problems is that I cannot get down to work when I should. *(Reverse scored)*

[all_gse02]

1. If I can’t do a job the first time, I keep trying until I can.

[all_gse03]

1. When I set important goals for myself, I rarely achieve them. *(Reverse scored)*

[all_gse04]

1. I give up on things before completing them. *(Reverse scored)*

[all_gse05]

1. I avoid facing difficulties. *(Reverse scored)*

[all_gse06]

1. If something looks too complicated, I will not even bother to try it. *(Reverse scored)*

[all_gse07]

1. When I have something unpleasant to do, I stick to it until I finish it.

[all_gse08]

1. When I decide to do something, I go right to work on it.

[all_gse09]

1. When trying to learn something new, I soon give up if I am not initially successful. *(Reverse scored)*

[all_gse10]

1. When unexpected problems occur, I don’t handle them well. *(Reverse scored)*

[all_gse11]

1. I avoid trying to learn new things when they look too difficult for me. *(Reverse scored)*

[all_gse12]

1. Failure just makes me try harder.

[all_gse13]

1. I feel insecure about my ability to do things. *(Reverse scored)*

[all_gse14]

1. I am a self-reliant person.

[all_gse15]

1. I give up easily. *(Reverse scored)*

[all_gse16]

1. I do not seem capable of dealing with most problems that come up in life. *(Reverse scored)*

[all_gse17]

**HEXACO-60 Personality Inventory** (From Ashton & Lee, 2009)

On the following pages, you will find a series of statements about you. Please read each statement and decide how much you agree or disagree with that statement. Then indicate your response using the following scale:

5 = strongly agree

4 = agree

3 = neutral (neither agree nor disagree)

2 = disagree

1 = strongly disagree

Please answer every statement, even if you are not completely sure of your response.

1. I would be quite bored by a visit to an art gallery.

[all_hex01]

1. I plan ahead and organize things, to avoid scrambling at the last minute.

[all_hex02]

1. I rarely hold a grudge, even against people who have badly wronged me.

[all_hex03]

1. I feel reasonably satisfied with myself overall.

[all_hex04]

1. I would feel afraid if I had to travel in bad weather conditions.

[all_hex05]

1. I wouldn’t use flattery to get a raise or promotion at work, even if I thought it would succeed.

[all_hex06]

1. I’m interested in learning about the history and politics of other countries.

[all_hex07]

1. I often push myself very hard when trying to achieve a goal.

[all_hex08]

1. People sometimes tell me that I am too critical of others.

[all_hex09]

1. I rarely express my opinions in group meetings.

[all_hex10]

1. I sometimes can’t help worrying about little things.

[all_hex11]

1. If I knew that I could never get caught, I would be willing to steal a million dollars.

[all_hex12]

1. I would enjoy creating a work of art, such as a novel, a song, or a painting.

[all_hex13]

1. When working on something, I don’t pay much attention to small details.

[all_hex14]

1. People sometimes tell me that I’m too stubborn.

[all_hex15]

1. I prefer jobs that involve active social interaction to those that involve working alone.

[all_hex16]

1. When I suffer from a painful experience, I need someone to make me feel comfortable.

[all_hex17]

1. Having a lot of money is not especially important to me.

[all_hex18]

1. I think that paying attention to radical ideas is a waste of time.

[all_hex19]

1. I make decisions based on the feeling of the moment rather than on careful thought.

[all_hex20]

1. People think of me as someone who has a quick temper.

[all_hex21]

1. On most days, I feel cheerful and optimistic.

[all_hex22]

1. I feel like crying when I see other people crying.

[all_hex23]

1. I think that I am entitled to more respect than the average person is.

[all_hex24]

1. If I had the opportunity, I would like to attend a classical music concert.

[all_hex25]

1. When working, I sometimes have difficulties due to being disorganized.

[all_hex25]

1. My attitude toward people who have treated me badly is “forgive and forget.”

[all_hex27]

1. I feel that I am an unpopular person.

[all_hex28]

1. When it comes to physical danger, I am very fearful.

[all_hex29]

1. If I want something from someone, I will laugh at that person’s worst jokes.

[all_hex30]

1. I’ve never really enjoyed looking through an encyclopedia.

[all_hex31]

1. I do only the minimum amount of work needed to get by.

[all_hex32]

1. I tend to be lenient in judging other people.

[all_hex33]

1. In social situations, I’m usually the one who makes the first move.

[all_hex34]

1. I worry a lot less than most people do.

[all_hex35]

1. I would never accept a bribe, even if it were very large.

[all_hex36]

1. People have often told me that I have a good imagination.

[all_hex37]

1. I always try to be accurate in my work, even at the expense of time.

[all_hex38]

1. I am usually quite flexible in my opinions when people disagree with me.

[all_hex39]

1. The first thing that I always do in a new place is to make friends.

[all_hex40]

1. I can handle difficult situations without needing emotional support from anyone else.

[all_hex41]

1. I would get a lot of pleasure from owning expensive luxury goods.

[all_hex42]

1. I like people who have unconventional views.

[all_hex43]

1. I make a lot of mistakes because I don’t think before I act.

[all_hex44]

1. Most people tend to get angry more quickly than I do.

[all_hex45]

1. Most people are more upbeat and dynamic than I generally am.

[all_hex46]

1. I feel strong emotions when someone close to me is going away for a long time.

[all_hex47]

1. I want people to know that I am an important person of high status.

[all_hex48]

1. I don’t think of myself as the artistic or creative type.

[all_hex49]

1. People often call me a perfectionist.

[all_hex50]

1. Even when people make a lot of mistakes, I rarely say anything negative.

[all_hex51]

1. I sometimes feel that I am a worthless person.

[all_hex52]

1. Even in an emergency I wouldn’t feel like panicking.

[all_hex53]

1. I wouldn’t pretend to like someone just to get that person to do favors for me.

[all_hex54]

1. I find it boring to discuss philosophy.

[all_hex55]

1. I prefer to do whatever comes to mind, rather than stick to a plan.

[all_hex56]

1. When people tell me that I’m wrong, my first reaction is to argue with them.

[all_hex67]

1. When I’m in a group of people, I’m often the one who speaks on behalf of the group.

[all_hex58]

1. I remain unemotional even in situations where most people get very sentimental.

[all_hex59]

1. I’d be tempted to use counterfeit money, if I were sure I could get away with it.

[all_hex60]

Honesty-Humility: 6, 12R, 18, 24R, 30R, 36, 42R, 48R, 54, 60R

Emotionality: 5, 11, 17, 23, 29, 35R, 41R, 47, 53R, 59R
Extraversion: 4, 10R, 16, 22, 28R, 34, 40, 46R, 52R, 58
Agreeableness (versus Anger): 3, 9R, 15R, 21R, 27, 33, 39, 45, 51, 57R

Conscientiousness: 2, 8, 14R, 20R, 26R, 32R, 38, 44R, 50, 56R

Openness to Experience: 1R, 7, 13, 19R, 25, 31R, 37, 43, 49R, 55R
(R indicates reverse-scored item.)

**Self-Monitoring Scale** (From Lennox & Wolfe, 1984)

*Each of the following questions is posed using a 5-point scale anchored by response options Strongly Agree and Strongly Disagree.*

1. In social situations, I have the ability to alter my behavior if I feel that something else is called for.

[all_sm01]

1. I am often able to read people’s true emotions correctly through their eyes.

[all_sm02]

1. I have the ability to control the way I come across to people, depending on the impression I wish to give them.

[all_sm03]

1. In conversations, I am sensitive to even the slightest change in the facial expression of the person I’m conversing with.

[all_sm04]

1. My powers of intuition are quite good when it comes to understanding others’ emotions and motives.

[all_sm05]

1. I can usually tell when others consider a joke to be in bad taste, even though they may laugh convincingly.

[all_sm06]

1. When I feel that the image I am portraying isn’t working, I can readily change it to something that does.

[all_sm07]

1. I can usually tell when I’ve said something inappropriate by reading it in the listener’s eyes.

[all_sm08]

1. I have trouble changing my behavior to suit different people and different situations. *(Reverse scored)*

[all_sm09]

1. I have found that I can adjust my behavior to meet the requirements of any situation I find myself in.

[all_sm10]

1. If someone is lying to me, I usually know it at once from that person’s manner of expression.

[all_sm11]

1. Even when it might be to my advantage, I have difficulty putting up a good front. *(Reverse scored)*

[all_sm12]

1. Once I know what the situation calls for, it’s easy for me to regulate my actions accordingly.

[all_sm13]

*Items 1, 3, 7, 9, 10, 12, and 13 form the “Ability to modify self-presentation” subscale. Items 2, 4, 5, 6, 8, and 11 form the “Sensitivity to expressive behavior of others” subscale.*

**Rosenberg Self-Esteem Scale** (From Rosenberg, 1979)

*Each of the following questions is posed using a 4-point scale anchored by response options Strongly agree and Strongly disagree.*

Please record the appropriate answer for each item, depending on whether you strongly agree, agree, disagree, or strongly disagree with it.

1. On the whole, I am satisfied with myself.

[all_rse01]

1. At times I think I am no good at all. *(Reverse scored)*

[all_rse02]

1. I feel that I have a number of good qualities.

[all_rse03]

1. I am able to do things as well as most other people.

[all_rse04]

1. I feel I do not have much to be proud of. *(Reverse scored)*

[all_rse05]

1. I certainly feel useless at times. *(Reverse scored)*

[all_rse06]

1. I feel that I’m a person of worth.

[all_rse07]

1. I wish I could have more respect for myself. *(Reverse scored)*

[all_rse08]

1. All in all, I am inclined to think that I am a failure. *(Reverse scored)*

[all_rse09]

1. I take a positive attitude toward myself.

[all_rse10]

**Rejection Sensitivity: Adult** (From Berensen et al., 2009)

The items below describe situations in which people sometimes ask things of others. For each time, **imagine that you are in the situation, and then answer the questions that follow it.**

1. You ask your parents or another family member for a loan to help you through a difficult financial time.
   1. How concerned or anxious would you be over whether or not your family would want to help you? *(6-point; very unconcerned-very concerned)*

[all_rsa1a]

- 1. I would expect that they would agree to help as much as they can. *(6-point; very unlikely-very likely)*

[all_rsa1b]

1. You approach a close friend to talk after doing or saying something that seriously upset him/her.
   1. How concerned or anxious would you be over whether or not your friend would want to talk with you? *(6-point; very unconcerned-very concerned)*

[all_rsa2a]

- 1. I would expect that he/she would want to talk with me to try to work things out. *(6-point; very unlikely-very likely)*

[all_rsa2b]

1. You bring up the issue of sexual protection with your significant other and tell him/her how important you think it is.
   1. How concerned or anxious would you be over his/her reaction? *(6-point; very unconcerned-very concerned)*

[all_rsa3a]

- 1. I would expect he/she would be willing to discuss our possible options without getting defensive. *(6-point; very unlikely-very likely)*

[all_rsa3b]

1. You ask your supervisor for help with a problem you have been having at work.
   1. How concerned or anxious would you be over whether or not the person would want to help you? *(6-point; very unconcerned-very concerned)*

[all_rsa4a]

- 1. I would expect that they would want to try to help me out. *(6-point; very unlikely-very likely)*

[all_rsa4b]

1. After a bitter argument, you call or approach your significant other because you want to make up.
   1. How concerned or anxious would you be over whether or not your significant other would want to make up with you? *(6-point; very unconcerned-very concerned)*

[all_rsa5a]

- 1. I would expect that he/she would be at least as eager to make up as I would be. *(6-point; very unlikely-very likely)*

[all_rsa5b]

1. You ask your parents or other family members to come to an occasion important to you.
   1. How concerned or anxious would you be over whether or not they would want to come? *(6-point; very unconcerned-very concerned)*

[all_rsa6a]

- 1. I would expect that they would want to come. *(6-point; very unlikely-very likely)*

[all_rsa6b]

1. At a party, you notice someone on the other side of the room that you’d like to get to know, and you approach him or her to try to start a conversation.
   1. How concerned or anxious would you be over whether or not the person would want to talk with you? *(6-point; very unconcerned-very concerned)*

[all_rsa7a]

- 1. I would expect that he/she would want to talk with me. *(6-point; very unlikely-very likely)*

[all_rsa7b]

1. Lately you’ve been noticing some distance between yourself and your significant other, and you ask him/her if there is something wrong.
   1. How concerned or anxious would you be over whether or not he/she still loves you and wants to be with you? *(6-point; very unconcerned-very concerned)*

[all_rsa8a]

- 1. I would expect that he/she will show sincere love and commitment to our relationship no matter what else may be going on. *(6-point; very unlikely-very likely)*

[all_rsa8b]

1. You call a friend when there is something on your mind that you feel you really need to talk about.
   1. How concerned or anxious would you be over whether or not your friend would want to listen? *(6-point; very unconcerned-very concerned)*

[all_rsa9a]

- 1. I would expect that he/she would listen and support me. *(6-point; very unlikely-very likely)*

[all_rsa9b]

**Satisfaction with Life Scale** (From Diener et al., 1985)

*Each of the following questions is posed using a 7-point scale anchored by response options Strongly Agree and Strongly Disagree.*

Below are five statement with which you may agree or disagree. Using the 1-7 scale below, indicate your agreement with each item. Please be open and honest in your responding.

1. In most ways my life is close to my ideal.

[all_swl1]

1. The conditions of my life are excellent.

[all_swl2]

1. I am satisfied with my life.

[all_swl3]

1. So far I have gotten the important things I want in my life.

[all_swl4]

1. If I could live my life over, I would change almost nothing.

[all_swl5]

**Emotion Regulation Questionnaire** (Gross & John, 2003)

*Each of the following questions is posed using a 7-point scale anchored by response options Strongly Agree and Strongly Disagree.*

Reappraisal factor:

1. I control my emotions by changing the way I think about the situation I’m in.

[all_er01]

1. When I want to feel less negative emotion, I change the way I’m thinking about the situation.

[all_er02]

1. When I want to feel more positive emotion, I change the way I’m thinking about the situation.

[all_er03]

1. When I want to feel more positive emotion (such as joy or amusement), I change what I’m thinking about.

[all_er04]

1. When I want to feel less negative emotion (such as sadness or anger), I change what I’m thinking about.

[all_er05]

1. When I’m faced with a stressful situation, I make myself think about it in a way that helps me stay calm.

[all_er06]

Suppression factor:

1. I control my emotions by not expressing them.

[all_er07]

1. When I am feeling negative emotions, I make sure not to express them.

[all_er08]

1. I keep my emotions to myself.

[all_er09]

1. When I am feeling positive emotions, I am careful not to express them.

[all_er10]

**Authenticity Scale** (From Wood et al., 2008)

*Each of the following questions is posed using a 7-point scale anchored by response options Does not describe me at all to Describes me very well.*

1. I think it is better to be yourself, than to be popular.

[all_auth01]

1. I don’t know how I really feel inside.

[all_auth02]

1. I am strongly influenced by the opinions of others.

[all_aut03]

1. I usually do what other people tell me to do.

[all_auth04]

1. I always feel I need to do what others expect me to do.

[all_auth05]

1. Other people influence me greatly.

[all_auth06]

1. I feel as if I don’t know myself very well.

[all_auth07]

1. I always stand by what I believe in.

[all_auth08]

1. I am true to myself in most situations.

[all_auth09]

1. I feel out of touch with the “real me”.

[all_auth10]

1. I live in accordance with my values and beliefs.

[all_auth11]

1. I feel alienated from myself.

[all_auth12]

**Belonging Uncertainty** (Adapted from Walton & Cohen, 2007)

*Each of the following questions is posed using a 7-point scale anchored by response options Strongly Agree and Strongly Disagree.*

1. Sometimes I feel that I belong, and sometimes I feel that I don’t belong.

[all_bu1]

1. When something bad happens, I feel that maybe I don’t belong.

[all_bu2]

1. When something good happens, I feel that I really belong.

[all_bu3]

**Final Instructions**

Thank you for participating! You have now completed the study. Please alert the researcher that you have finished.
